# Supplementary material for: The influence of Holliday junction sequence and dynamics on DNA crystal self-assembly
Source: Nat Commun. 2022 Jun 3;13:3112. doi: 10.1038/s41467-022-30779-6 (PMC9166708; doi:10.1038/s41467-022-30779-6)
Supplement: Supplementary file 1 — Supplementary Information [file 41467_2022_30779_MOESM1_ESM.pdf]

Supplementary Information

The influence of Holliday junction sequence and dynamics on DNA crystal self-assembly

Chad R. Simmons[1†], Tara MacCulloch[1,2†], Miroslav Krepl[3,4], Michael Matthies[1], Alex Buchberger[1,2], Ilyssa Crawford[1,2], Jiří Šponer[3,4], Petr Šulc[1,2], Nicholas Stephanopoulos[1,2*] & Hao Yan[1,2*]

Biodesign Center for Molecular Design and Biomimetics, 1001 S. McAllister Ave. Tempe, AZ 85287, Arizona State University (USA)[1]

School of Molecular Sciences, Arizona State University, Tempe, AZ 85287 (USA)[2]

Institute of Biophysics of the Czech Academy of Sciences, Krlovopolska 135, 612 65 Brno, Czech Republic[3]

Regional Centre of Advanced Technologies and Materials, Czech Advanced Technology and Research Institute (CATRIN), Palacky University Olomouc, Slechtitelu 241/27,783 71, Olomouc Holice, Czech Republic[4]

[†]These authors contributed equally

[*]To whom correspondence should be addressed

hao.yan@asu.edu

nstepha1@asu.edu

Supplementary Information Table of Contents

Supplementary Figure 1. Representative crystal structures of junction and duplexes **3**

Supplementary Table 1. Data collection and refinement statistics **4-15**

Supplementary Figure 2. Structural representations of junction and duplex models **16**

Supplementary Figure 3. 2D topologies of the central building blocks containing Holliday junctions ... **17**

Supplementary Table 2. DNA sequences used for each constituent oligonucleotide **18-20**

Supplementary Table 3. Components for each of the 48 buffer conditions **21**

Supplementary Figure 4. Observed lattice packing from three unique space groups **22**

Supplementary Figure 5. Representative bright-field images of the 4x5 crystals **23**

Supplementary Table 4. The buffer conditions used for the corresponding images of the 4x5 crystals .. **24**

Supplementary Table 5. Unit cell dimensions for each corresponding 4x5 junction crystal **25**

Supplementary Figure 6. Superimposed 4x5 structures of representative $P3_221$ vs. $P3_2$ crystals **26**

Supplementary Table 6. Summary of the resulting space group and corresponding resolution for all 4x5 crystal structures **27**

Supplementary Figure 7. Symmetry related duplexes show the full crystal lattice for both symmetries observed in the 4x5 motif **28**

Supplementary Table 7. Calculated interduplex angles (IDA) corresponding to each junction structure for the 4x5 system **29**

Supplementary Figure 8. Representative bright-field images of the 4x6 crystals **30**

Supplementary Table 8. Summary of the resulting space group and corresponding resolution for all 4x6 crystal structures **31**

Supplementary Table 9. The buffer conditions used for the corresponding bright field images shown in Supplementary figure 9 for the 4x6 system **32**

Supplementary Figure 9. Bright field images for the five junctions resulting in $R3$ symmetry in the 4x6 crystals **33**

Supplementary Table 10. The buffer conditions used for the corresponding bright field images shown in Supplementary figure 10 for the 4x6 system comparing the R3 vs $P3_2$ preferences **33**

Supplementary Figure 10. Superimposed views of 4x6 junction crystals exhibiting both $P3_2$ and $R3$ symmetry **34**

Supplementary Table 11. Parallel comparison of the fate of each junction sequence between the 4x5 and 4x6 systems **34**

Supplementary Table 12. Unit cell dimensions for each corresponding 4x6 junction crystal **35**

Supplementary Figure 11. Symmetry related duplexes show the full crystal lattice for both symmetries observed in the 4x6 motif **36**

Supplementary Table 13. Calculated interduplex angles (IDA) corresponding to each junction structure for the 4x6 system **37**

Supplementary Figure 12. Duplex comparison of the original and scrambled 4x6 sequences **37**

Supplementary Figure 13. Representative bright-field images from the crystallization screen of all 36 immobile Holliday junctions in the 4x6 "scramble" system **38**

Supplementary Table 14. Buffers used for representative bright field image shown in supplementary figure 14 for the 4x6 scramble system **39**

Supplementary Table 15. Summary of the resulting space group and corresponding resolution for all 4x6 "scramble" crystal structures **40**

Supplementary Table 16. Parallel comparison of the fate of each junction sequence between the 4x5, 4x6, and 4x6 scrambled sequence systems **40**

Supplementary Figure 14. Stereoviews of superimposed structures representing unique or consistent symmetries between the 4x6 and 4x6 scrambled structures **41**

Supplementary Figure 15. Symmetry related duplexes show the full crystal lattice for both symmetries observed in the 4x6 "scramble" motif..**42**

Supplementary Table 17. Unit cell dimensions for each corresponding 4x6 scramble junction crystals .**43**

Supplementary Table 18. Calculated interduplex angles (IDA) corresponding to each junction structure for the 4x6 scrambled sequence system **44**

Supplementary Figure 16. Definition of ion positions 1 and 2 ....**44**

Supplementary Figure 17. Conserved binding sites are consistent regardless of crystal symmetry.........**45**

Supplementary Figure 18. Stereoviews of superimposed structures representing conserved ion binding sites in each system **46**

Supplementary Figure 19. Histograms of J_{twist} interhelical angle populations in MD simulations of all 36 immobile HJs in solution **47**

Supplementary Figure 20. Classical molecular interaction potential (CMIP) calculation.......................**48**

Supplementary Figure 21. Cacodylate ion in density **49**

Supplementary Table 19. Median values of the J_{twist} interhelical angles in MD simulations of all 36 immobile HJs **50**

Supplementary Figure 22. Unusual β/γ conformational states in selected MD simulation of J5 using the parmbsc1 DNA force field **51**

Supplementary Discussion 1. HJ simulations using the parmbsc1 DNA force field **52**

Supplementary Figure 23. Snapshot of the entire simulation cell of the J5 junction..............................**52**

Supplementary Figure 24. Comparison between standard- and extended-length MD simulations **53**

Supplementary Table 20. PDB accession codes for each deposited structure **54**

Supplementary References. **54**

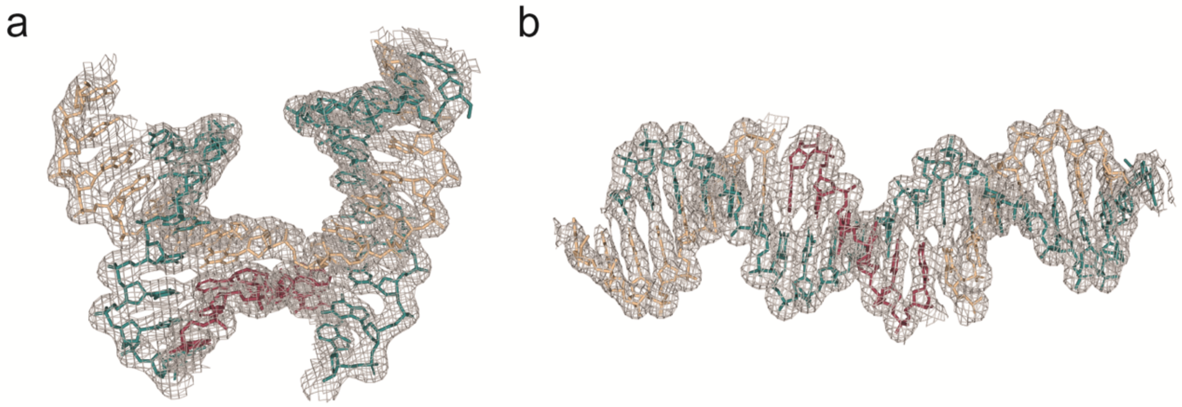

Supplementary Figure 1. Representative crystal structures of junction and duplexes. (a) The contents of the asymmetric unit was defined as a Holliday junction containing 10 and 11 bp (21 bp total) on each respective arm of the four-way branched junction. The structure is modeled into $2F_o - F_c$ density contoured at $\sigma = 1.5$. (b) Crystal structure of a 21 bp duplex for the identical sequence contained in (a) into electron density contoured at $\sigma = 1.5$. All component oligonucleotides are colored with the assignments described above.

Supplementary Table 1. Data collection and refinement statistics for all crystal structures, both junction and duplex models, across the three systems and 36 junctions.

**Variation (4X5)**	**J1**	**J3**	**J5**	**J6**	**J7**	**J8**	**J9**
PDB Code: Junction	6X8C	6XDV	6XDW	6XDX	6XDY	6XDZ	6XEI
PDB Code: Duplex	5KEK	6WQG	6WRB	6X8B	6WSN	6WSO	6WSP
**Data Collection**							
Beamline	NSLS X25	APS 19-ID	APS 19-BM	APS 19-ID	APS 19-ID	APS 19-ID	APS 19-ID
Space group	$P3_221$	$P3_2$	$P3_221$	$P3_2$	$P3_221$	$P3_2$	$P3_2$
Resolution (Å)	3.1	3	3.15	2.9	3.05	3.1	3.05
**Cell dimensions**							
a, b, c (Å)	67.9,67.9,59.3	68.9, 68.9,60.7	67.9,67.9,59.5	68.9,68.9,59.4	67.8,67.8,60.5	68.9,68.9,59.8	68.7,68.7,60.8
α, β, γ (°)	90,90,120	90,90,120	90,90,120	90, 90, 120	90, 90, 120	90, 90, 120	90, 90, 120
Wavelength (Å)	0.98	0.92	1.00	1.00	1.00	0.92	0.92
Total observations	29781	64132	35589	64266	53942	50597	57115
No. unique reflections	3041	6381	2891	6565	3237	5407	5886
R_{pim}	2.6(26.8)	3.2(21.2)	4.4(20.9)	4.4(35.4)	2.7 (27.0)	3.2(27.9)	3.1(25.1)
$CC_{1/2}$	1.001(0.888)	1.00(.938)	1.008(.882))	0.997(0.77)	1.007 (0.865)	0.857 (.856)	0.972(.901)
$I/\sigma I$	40.08(2.96)	45.828(1.85)	17.38(1.72)	42.1(1.0)	55.438 (1.22)	42.19 (1.571)	33.2(1.556)
Completeness (%)	99.4(93.5)	99.1(90.4)	98.0(82.8)	93.8(64.5)	99.6 (99.4)	94.0 (62.2)	95.9 (66.8)
Redundancy	9.8(7.0)	10.1(8.1)	12.3(7.3)	9.8 (8.0)	16.7 (12.4)	9.4 (7.6)	9.7(7.7)
**Refinement: Junction**							
R_{work}/R_{free}	23.84/26.71	22.67/25.24	22.40/25.99	22.40/25.99	23.97/25.13	22.16/23.11	24.55/26.74
**No. atoms**							
DNA	855	855	855	855	855	855	855
ligand/ion	1	3	1	1	0	2	3
**R.m.s deviations**							
Bond lengths (Å)	0.004	0.007	0.012	0.012	0.004	0.005	0.005
Bond angles (°)	0.604	0.765	1.231	1.231	0.611	0.641	0.669
**Refinement: Duplex**							
R_{work}/R_{free}	20.42/25.97	23.30/25.05	25.08/26.33	23.78/25.05	23.13/52.95	19.87/22.23	26.14/28.89
**No. atoms**							
DNA	853	856	855	855	855	855	855
ligand/ion	2	3	2	5	0	3	3
**R.m.s deviations**							
Bond lengths (Å)	0.0139	0.006	0.005	0.005	0.004	0.01	0.005
Bond angles (°)	1.317	0.82	0.609	0.727	0.582	0.962	0.71
*The value for the highest-resolution shell is shown in parentheses							

Variation (4X5)	J10	J14	J15	J16	J19	J20	J21
PDB Code: Junction	6XEJ	6XEK	6XEL	6XEM	6XFC	6XFD	6XFE
PDB Code: Duplex	6WSQ	6WSR	6WSS	6WST	6WSU	6WSV	6WSW
**Data Collection**							
Beamline	APS 19-ID	APS 19-ID	APS 19-ID	APS 19-ID	APS 19-ID	APS 19-ID	ALS 5.0.2
Space group	$P3_221$	$P3_221$	$P3_2$	$P3_2$	$P3_221$	$P3_221$	$P3_2$
Resolution (Å)	3.05	2.85	3	3.05	2.75	3.1	3.1
**Cell dimensions**							
a, b, c (Å)	68.8,68.8,62.0	68.9,68.9,62.1	68.8,68.8,60.9	69.0,69.0,61.3	68.5,68.5,60.8	67.6.67.6,60.4	69.0,69.0,59.4
α, β, γ (°)	90, 90, 120	90, 90, 120	90, 90, 120	90, 90, 120	90, 90, 120	90, 90, 120	90,90,120
Wavelength (Å)	0.98	0.92	0.92	0.92	0.98	0.98	0.92
Total observations	49240	80590	62192	54438	50811	54504	46129
No. unique reflections	4339	4213	6272	5999	4478	3074	4917
R_{pim}	2.7 (32.7)	1.9 (17.1)	2.9 (20.5)	4.0 (26.8)	3.6 (52.6)	3.3 (21.4)	2.2 (29.2)
$CC_{1/2}$	0.992 (0.817)	0.917(0.916)	0.896 (.925)	1.072 (.925)	0.957 (.652)	0.994 (0.946)	.990 (.825)
$I/\sigma I$	58.70(1.96)	66.19 (3.2)	40.88 (1.9)	33.0 (2.27)	54.91 (1.435)	56.70 (1.5)	35.4 (1.1667)
Completeness (%)	98.5 (100.0)	99.8 (100.0)	97.3 (73.7)	96.9 (75.9)	98.6 (99.6)	99.3 (95.5)	87.3 (50.6)
Redundancy	11.3 (11.7)	19.1 (19.4)	9.9 (8.4)	9.1 (7.0)	11.3 (9.3)	17.7 (11.8)	9.4(6.4)
**Refinement: Junction**							
R_{work}/R_{free}	23.20/24.55	24.10/26.27	25.19/28.79	22.49/25.39	21.82/24.23	22.31/28.58	20.85/23.34
**No. atoms**							
DNA	855	855	855	855	855	855	856
ligand/ion	2	2	1	1	3	2	2
**R.m.s deviations**							
Bond lengths (Å)	0.011	0.011	0.005	0.006	0.006	0.004	0.005
Bond angles (°)	1.259	1.843	0.608	0.766	0.771	0.604	0.662
**Refinement: Duplex**							
R_{work}/R_{free}	24.30/29.19	23.42/24.47	24.83/27.21	21.29/26.26	21.09/23.28	24.34/28.26	23.39/27.79
**No. atoms**							
DNA	855	855	855	855	855	855	854
ligand/ion	1	2	2	3	4	0	3
**R.m.s deviations**							
Bond lengths (Å)	0.007	0.015	0.005	0.01	0.009	0.009	0.004
Bond angles (°)	0.859	1.422	0.633	0.994	0.992	1.448	0.66
*The value for the highest-resolution shell is shown in parentheses							

**Variation (4X5)**	**J22**	**J23**	**J24**	**J25**	**J26**	**J28**	**J29**
PDB Code: Junction	6XFF	6XFG	6XFW	6XGM	6XFX	6XFY	6XGZ
PDB Code: Duplex	6WSX	6WSY	6WSZ	6WT0	6WRJ	6WRI	6WT1
**Data Collection**							
Beamline	ALS 5.0.2	ALS 5.0.2	ALS 5.0.2	APS 19-ID	ALS 5.0.2	ALS 5.0.2	ALS 5.0.2
Space group	$P3_2$	$P3_221$	$P3_2$	$P3_2$	$P3_221$	$P3_2$	$P3_2$
Resolution (Å)	3.1	3.05	3.1	3.1	3.1	3.05	3.1
**Cell dimensions**							
a, b, c (Å)	69.4,69.4,59.4	68.5,68.5,60.2	68.5,68.5,60.2	69.0,69.0,59.5	67.6,67.6,60.6	69.0,69.0,60.6	68.7,68.7,58.1
α, β, γ (°)	90,90,120	90,90,120	90,90,120	90,90,120	90,90,120	90,90,120	90,90,120
Wavelength (Å)	0.92	1	1	1	1	1	1
Total observations	44428	61900	47136	44780	49300	56694	43300
No. unique reflections	4852	3313	5010	5321	2902	5829	4640
R_{pim}	4.3(34.3)	1.3(23.2)	2.6 (25.2)	8.1 (45.9)	2.0(8.3)	2.1(31.6)	5.3(18.2)
$CC_{1/2}$	.974(.718)	.999(.905)	0.977 (0.864)	0.961 (0.822)	1.009(.991)	.988(.855)	1.164(.943)
$I/\sigma I$	31.9 (1.163)	51.48(2.0)	36.08 (1.27)	28.94 (1.51)	36.17 (3.36)	29.37(1.357)	22.38(1.769)
Completeness (%)	85.1 (51.2)	99.9(99.4)	85.1 (47.9)	93.1 (65.7)	96.3(66.9)	95.2 (64.6)	84.3(51.3)
Redundancy	9.2 (6.4)	18.7 (15.9)	9.4 (7.1)	8.4 (5.0)	17.0 (13.1)	9.7 (7.2)	9.3 (7.1)
**Refinement: Junction**							
R_{work}/R_{free}	22.91/26.69	21.46/24.05	24.31/25.47	21.90/23.46	22.65/27.07	22.69/24.79	24.41/26.22
**No. atoms**							
DNA	855	855	855	855	855	855	855
ligand/ion	0	2	2	2	0	3	2
**R.m.s deviations**							
Bond lengths (Å)	0.006	0.006	0.004	0.008	0.005	0.005	0.005
Bond angles (°)	0.808	0.829	0.667	1.602	0.724	0.664	0.673
**Refinement: Duplex**							
R_{work}/R_{free}	24.61/25.63	21.78/25.45	23.79/26.17	24.73/26.23	23.49/28.15	19.75/24.75	21.78/25.54
**No. atoms**							
DNA	855	855	855	855	855	855	855
ligand/ion	0	2	3	2	0	2	2
**R.m.s deviations**							
Bond lengths (Å)	0.011	0.007	0.753	0.005	0.005	0.009	0.008
Bond angles (°)	1.072	0.005	0.602	0.668	0.692	0.996	1.467
*The value for the highest-resolution shell is shown in parentheses							

**Variation (4X5)**	**J31**	**J32**	**J33**	**J34**	**J35**	**J36**
PDB Code: Junction	6XG0	6XGJ	6XGN	6XGO	6XGK	6XGL
PDB Code: Duplex	6WRC	6WR9	6WR7	6WRA	6WR5	6WR3
**Data Collection**						
Beamline	ALS 5.0.2	ALS 5.0.2	ALS 5.0.2	APS 19-ID	ALS 5.0.2	ALS 5.0.2
Space group	$P3_2$	$P3_2$	$P3_2$	$P3_2$	$P3_2$	$P3_2$
Resolution (Å)	3.15	3.05	3.1	3.0	3.05	3.15
**Cell dimensions**						
a, b, c (Å)	68.9,68.9,59.0	69.0,69.0,60.8	68.4,68.4,61.30	68.8,68.8,59.7	68.7, 68.7, 60.1	68.8,68.8,60.6
α, β, γ (°)	90,90,120	90,90,120	90,90,120	90, 90, 120	90,90,120	90, 90, 120
Wavelength (Å)	1	0.92	1	1	1	0.92
Total observations	42070	51714	40438	36218	52784	41936
No. unique reflections	4650	5625	4430	6287	5471	4722
R_{pim}	3.8(16.6)	2.3(17.7)	2.6 (27.9)	5.9 (34.3)	2.3(12.9)	4.2(15.4)
$CC_{1/2}$	.981(.947)	1.015(.953)	.998 (0.605)	0.911 (.915)	.985(.970)	.996(.930)
$I/\sigma I$	35.59(2.11)	29.12 (1.739)	30.84 (1.16)	9.578(2.114)	36.06(3.381)	26.66 (1.7)
Completeness (%)	88.3 (62.2)	93.4 (72.8)	78.1 (32.3)	99.4 (95.6)	91.9(62.1)	87.3(55.0)
Redundancy	9.0 (5.6)	9.2 (6.3)	9.1 (6.2)	5.8 (4.7)	9.6 (8.0)	8.9 (5.6)
**Refinement: Junction**						
R_{work}/R_{free}	23.88/26.14	22.44/25.31	20.09/22.85	21.12/23.34	19.65/21.08	21.72/23.96
**No. atoms**						
DNA	855	855	855	855	855	855
ligand/ion	2	3	0	2	2	3
**R.m.s deviations**						
Bond lengths (Å)	0.004	0.008	0.008	0.011	0.015	0.007
Bond angles (°)	0.657	0.894	1.322	1.76	1.463	0.806
**Refinement: Duplex**						
R_{work}/R_{free}	24.82/26.72	22.28/24.63	20.62/24.21	20.64/23.51	25.04/26.36	24.75/25.52
**No. atoms**						
DNA	855	855	855	855	856	855
ligand/ion	0	3	0	2	2	3
**R.m.s deviations**						
Bond lengths (Å)	0.006	0.009	0.007	0.011	0.005	0.005
Bond angles (°)	0.74	0.985	1.426	1.732	0.638	0.71
*The value for the highest-resolution shell is shown in parentheses						

Variation (4X6)	J1	J2	J5	J7	J8
PDB Code: Junction	6XNA	7JFT	7JFU	7JFV	6XO5
PDB Code: Duplex	5VY6	7JPB	7JPA	7JPC	7JP9
**Data Collection**					
Beamline	ALS 8.2.2	NSLS 17-ID2	ALS 5.0.2	ALS 5.0.2	ALS 8.2.2
Resolution (Å)	3.05	3.15	3.15	3.1	3
Space group	$P3_2$	$P3_2$	$P3_2$	$P3_2$	$P3_2$
**Cell dimensions**					
a, b, c (Å)	68.44,68.44,55.68	69.11,69.11,56.40	68.17,68.17,55.46	68.01,68.01,54.15	68.30,68.30,54.28
α, β, γ (°)	90,90,120	90,90,120	90,90,120	90,90,120	90,90,120
Wavelength (Å)	1	0.98	1	0.92	1
Total observations	36158	27398	29992	17407	
No. unique reflections	4784	4138	3637	3716	3906
R_{pim}	4.5 (37.2)	4.8 (16.2)	3.6 (11.3)	3.7 (27.4)	4.0(27.9)
$CC_{1/2}$	0.945 (0.883)	0.991 (0.960)	1.066 (0.928)	0.978 (0.875)	0.983(0.87)
$I/\sigma I$	23.9 (1.9)	15.97 (2.33)	30.58 (3.0)	25.96 (1.29)	20.5 (0.79)
Completeness (%)	86.6 (55.7)	80.1 (41.5)	73.2 (36.3)	73.2 (34.4)	80.0 (44.3)
Redundancy	7.6 (6.7)	6.6 (3.2)	8.2 (6.2)	4.7(3.7)	4.6 (2.4)
**Refinement: Junction**					
R_{work}/R_{free}	20.17/23.42	22.42/24.68	21.32/26.32	19.27/23.44	21.19/24.68
**No. atoms**					
DNA	855	855	855	855	855
ligand/ion	4	2	0	0	1
**R.m.s deviations**					
Bond lengths (Å)	0.004	0.006	0.006	0.006	0.007
Bond angles (°)	0.646	0.773	0.759	0.776	1.551
**Refinement: Duplex**					
R_{work}/R_{free}	21.13/23.66	22.36/25.21	20.1/21.62	18.00/21.32	25.69/27.33
**No. atoms**					
DNA	851	855	855	855	855
ligand/ion	0	2	0	0	2
**R.m.s deviations**					
Bond lengths (Å)	0.07	0.005	0.007	0.007	0.006
Bond angles (°)	0.923	0.755	1.244	1.169	0.734
*The value for the highest-resolution shell is shown in parentheses					

Variation (4X6)	J10	J16	J20	J22	J23
PDB Code: Junction	7JFW	7JFX	7JH8	7JH9	7JHA
PDB Code: Duplex	7JP8	7JP7	7JP6	7JP5	7JON
**Data Collection**					
Beamline	ALS 5.0.2	APS 19-ID	APS 19-BM	APS 19-BM	ALS 8.2.2
Resolution (Å)	3.1	3.2	3.1	3.1	3.1
Space group	$P3_2$	$P3_2$	$P3_2$	$P3_2$	$P3_2$
**Cell dimensions**					
a, b, c (Å)	67.74,67.74,53.48	68.15.68.15,53.79	68.07.68.07,56.06	68.55,68.55,55.36	68.63,68.63,55.96
α, β, γ (°)	90,90,120	90,90,120	90,90,120	90,90,120	90,90,120
Wavelength (Å)	0.98	0.92	1	1.1	0.98
Total observations	23461	30431	34237	22626	29383
No. unique reflections	4054	3636	4830	4420	4641
R_{pim}	4.6 (26.5)	2.9 (10.3)	2.6 (14.8)	6.6 (46.2)	5.0 (22.9)
$CC_{1/2}$	0.98 (0.892)	0.879 (0.948)	0.993 (0.958)	0.998	1.009(0.908)
$I/\sigma I$	23.96 (1.57)	21.66 (3.92)	32.41 (2.64)	20.81 (1.6)	15.43 (1.6)
Completeness (%)	82.1 (47.6)	79.0 (43.9)	90.3 (62.5)	83.2 (48.9)	86.8 (56.6)
Redundancy	5.8 (4.9)	8.4 (4.2)	7.1 (5.6)	5.1 (4.1)	6.3 (4.7)
**Refinement: Junction**					
R_{work}/R_{free}	19.22/21.73	24.25/28.00	24.61/27.24	23.81/25.80	51.5/22.6
**No. atoms**					
DNA	855	855	858	855	855
ligand/ion	0	0	0	2	2
**R.m.s deviations**					
Bond lengths (Å)	0.006	0.006	0.006	0.005	0.006
Bond angles (°)	0.809	1.119	0.663	0.694	0.798
**Refinement: Duplex**					
R_{work}/R_{free}	22.82/24.73	24.23/25.96	26.05/27.00	24.43/26.86	22.72/24.41
**No. atoms**					
DNA	855	855	855	855	855
ligand/ion	1	3	1	2	5
**R.m.s deviations**					
Bond lengths (Å)	0.005	0.005	0.006	0.005	0.005
Bond angles (°)	0.701	0.802	0.691	0.798	0.729
*The value for the highest-resolution shell is shown in parentheses					

Variation (4X6)	J24	J26	J28	J30	J31	J33
PDB Code: Junction	7JHB	7JHC	6XO6	6XO7	6XO8	6XO9
PDB Code: Duplex	7JOL	7JOK	7JOJ	7JOI	7JOH	7JOG
**Data Collection**						
Beamline	APS 19-BM	APS 19-BM	NSLS 17-ID1	ALS 8.2.2	APS 19-ID	ALS 8.2.2
Resolution (Å)	3.1	3.1	3.1	3.15	4.2	3.1
Space group	$P3_2$	$P3_2$	$P3_2$	$P3_2$	$P3_2$	$P3_2$
**Cell dimensions**						
a, b, c (Å)	68.10,68.10,57.30	68.17,68.17,55.08	67.90,67.90,59.51	68.1,68.14,52.77	68.76,68.76,55.21	68.39,68.39,60.35
α, β, γ (°)	90,90,120	90,90,120	90,90,120	90,90,120	90,90,120	90,90,120
Wavelength (Å)	1.1	1	0.92		0.92	1
Total observations	26695	32651	43008	16727	15873	23644
No. unique reflections	4353	4614	4995	3159	2053	4538
R_{pim}	4.7 (26.8)	3.8 (21.2)	5.4 (20.0)	6.2 (27.6)	3.3 (33.6)	3.0 (19.4)
$CC_{1/2}$	0.997 (0.861)	0.994 (0.962)	? (0.962)	0.809 (0.841)	1.004 (0.753)	1.013 (0.92)
$I/\sigma I$	19.39 (1.38)	23.06 (2.75)	19.56 (2.33)	20.65 (1.33)	29.88 (1.36)	30.95 (1.65)
Completeness (%)	81.8 (50.0)	89.7 (57.8)	90.3 (63.5)	63.9 (25.3)	95.7 (80.6)	79.0 (38.5)
Redundancy	6.1 (4.0)	7.1 (5.7)	8.6 (6.1)	5.3 (4.0)	7.7 (7.3)	5.2 (3.9)
**Refinement: Junction**						
R_{work}/R_{free}	27.09/29.81	26.41/27.74	18.83/22.09	21.53/24.01	16.68/18.35	23.59/24.35
**No. atoms**						
DNA	855	855	855	855	855	855
ligand/ion	2	2	1	4	0	1
**R.m.s deviations**						
Bond lengths (Å)	0.005	0.005	0.01	0.005	0.007	0.01
Bond angles (°)	0.621	0.678	0.998	0.751	0.809	1
**Refinement: Duplex**						
R_{work}/R_{free}	24.76/25.72	24.6/26.74	19.31/22.30	24.78/26.79	20.49/23.90	22.47/25.74
**No. atoms**						
DNA	853	851	855	855	855	855
ligand/ion	3	3	1	2	0	1
**R.m.s deviations**						
Bond lengths (Å)	0.007	0.005	0.007	0.004	0.004	0.008
Bond angles (°)	0.868	0.766	0.735	0.695	0.621	0.926
*The value for the highest-resolution shell is shown in parentheses						

Variation (4X6)	J4	J5	J31	J33	J36
PDB Code: Junction	7JHR	7JHS	7JHT	7JHU	7JHV
PDB Code: Duplex	7HRY	7JRZ	7JS0	7JS1	7JS2
**Data Collection**					
Beamline	ALS 5.0.2	APS 19-ID	ALS 5.0.2	APS 19-ID	ALS 5.0.2
Resolution (Å)	3.15	3.1	3.15	3.15	3.05
Space group	R3	R3	R3	R3	R3
**Cell dimensions**					
a, b, c (Å)	115.15,115.15,48.66	114.78,114.78,49.62	116.08,116.08,49.43	113.87,113.87,50.81	114.61,114.61,50.35
α, β, γ (°)	90,90,120	90,90,120	90,90,120	90,90,120	90,90,120
Space group	H3	H3	H3	H3	H3
**Cell dimensions**					
a, b, c (Å)	64.95	68.22	68.26	67.86	68.11
α, β, γ (°)	113.9	114.37	114.55	113.99	114.17
Wavelength (Å)	1	0.92	1	0.92	1
Total observations	28824	38645	28107	25502	45010
No. unique reflections	3297	4170	3347	3676	4633
R_{pim}	2.0 (10.9)	3.8 (21.6)	4.0 (18.7)	5.8 (21.0)	4.2 (24.1)
$CC_{1/2}$	1.019 (0.967)	0.972 (0.955)	0.948 (0.899)	0.905 (0.867)	0.924 (0.908)
$I/\sigma I$	35.85 (2.71)	11.78 (1.5)	27.18 (1.83)	40.30 (2.03)	40.82 (1.69)
Completeness (%)	79.4 (26.6)	94.0 (65.8)	81.1 (30.9)	86.4 (35.3)	99.4 (95.4)
Redundancy	8.7 (6.7)	9.3 (6.0)	8.4 (5.2)	6.9 (3.7)	9.7(8.2)
**Refinement: Junction**					
R_{work}/R_{free}	24.54/27.75	25.92/26.87	22.17/25.84	24.36/27.27	20.79/23.61
**No. atoms**					
DNA	855	855	855	849	855
ligand/ion	2	3	1	0	0
**R.m.s deviations**					
Bond lengths (Å)	0.004	0.005	0.005	0.005	0.007
Bond angles (°)	0.635	0.74	0.679	0.768	0.797
**Refinement: Duplex**					
R_{work}/R_{free}	25.06/28.32	25.97/29.21	20.14/21.68	22.09/26.33	18.81/22.12
**No. atoms**					
DNA	855	855	855	855	855
ligand/ion	1	4	2	0	0
**R.m.s deviations**					
Bond lengths (Å)	0.005	0.006	0.012	0.006	0.014
Bond angles (°)	0.661	0.674	1.211	0.767	1.246

*The value for the highest-resolution shell is shown in parentheses

**Variation (4X6 Scramble)**	**J1**	**J2**
PDB Code: Junction	7JK0	7JJZ
PDB Code: Duplex	7JKD	7JKE
**Data Collection**		
Beamline	ALS 5.0.2	ALS 5.0.2
Resolution (Å)	3.05	3.05
Space group	$P3_2$	$P3_2$
**Cell dimensions**		
a, b, c (Å)	67.97,67.97,55.83	68.27,68.27,55.79
α, β, γ (°)	90,90,120	90,90,120
Wavelength (Å)	1	1
Total observations	23080	36297
No. unique reflections	4289	4231
R_{pim}	3.2 (21.3)	3.7 (30.7)
$CC_{1/2}$	1.027 (0.951)	0.981 (0.785)
$I/\sigma I$	30.28(2.31)	21.33 (1.125)
Completeness (%)	78.6 (49.1)	77.7 (46.1)
Redundancy	5.4 (5.1)	8.6 (7.5)
**Refinement: Junction**		
R_{work}/R_{free}	22.46/24.75	23.81/26.46
**No. atoms**		
DNA	855	855
ligand/ion	1	0
**R.m.s deviations**		
Bond lengths (Å)	0.004	0.005
Bond angles (°)	0.631	0.73
**Refinement: Duplex**		
R_{work}/R_{free}	23.62/27.00	25.15/27.49
**No. atoms**		
DNA	855	855
ligand/ion	1	3
**R.m.s deviations**		
Bond lengths (Å)	0.004	0.004
Bond angles (°)	0.597	0.563
*The value for the highest-resolution shell is shown in parentheses		

Variation (4X6 Scramble)	J3	J5	J7	J8	J10	J14
PDB Code: Junction	7JJY	7JJX	7JJW	7JJ6	7JJ5	7JJ4
PDB Code: Duplex	7JKG	7JKH	7JKI	7JKJ	7JKK	7JL9
**Data Collection**						
Beamline	ALS 5.0.2	ALS 5.0.2	APS 19-ID	ALS 5.0.2	ALS 5.0.2	ALS 5.0.2
Resolution (Å)	2.85	3.1	3	3.05	2.8	3
Space group	R3	R3	R3	R3	R3	R3
**Cell dimensions**						
a, b, c (Å)	112.21,112.21,50.99	113.26,113.26,49.90	113.72,113.72,52.05	113.17,113.17,50.78	113.08,113.08,52.13	112.75,112.75,51.06
α, β, γ (°)	90,90,120	90,90,120	90,90,120	90,90,120	90,90,120	90,90,120
Space group	H3	H3	H3	H3	H3	H3
**Cell dimensions**						
a, b, c (Å)	66.94	67.33	67.44	66.97	67.66	66.82
α, β, γ (°)	113.9	114.14	113.69	114.05	113.63	113.89
Wavelength (Å)	1	1	1	1	1	1
Total observations	57963	33207	45718	36594	62932	45460
Unique reflections	5596	3640	4711	4101	6150	4615
R_{pim}	2.8 (35.5)	2.0 (17.3)	4.6 (26.2)	5.4 (27.8)	3.8 (38.8)	2.1 (29.9)
$CC_{1/2}$	0.986 (0.82)	1.012 (0.902)	0.951 (0.894)	0.933 (0.807)	1.069 (0.893)	1.001 (0.833)
$I/\sigma I$	44 (1.5)	36.13(2.04)	51.26 (2.36)	22.17 (1.15)	57.96 (1.35)	43.75 (1.0)
Completeness (%)	100 (100)	84.7 (39.3)	95.5 (89.6)	91.7 (54.1)	99.7 (100)	97.5 (76.7)
Redundancy	10.4 (9.2)	9.1 (7.4)	9.7 (7.1)	8.9 (6.0)	10.2 (9.4)	9.9 (7.4)
**Refinement: Junction**						
R_{work}/R_{free}	20.93/24.29	20.07/23.03	21.04/25.18	19.86/23.74	22.70/24.30	22.60/26.75
**No. atoms**						
DNA	855	855	855	855	855	855
ligand/ion	2	2	2	2	1	2
**R.m.s deviations**						
Bond lengths (Å)	0.008	0.006	0.005	0.008	0.005	0.005
Bond angles (°)	0.946	0.755	0.689	0.885	0.784	0.701
**Refinement: Duplex**						
R_{work}/R_{free}	20.86/23.42	24.45/25.41	21.44/25.79	22.85/24.08	23.02/25.68	22.41/25.48
**No. atoms**						
DNA	855	855	855	855	855	855
ligand/ion	4	0	2	2	4	2
**R.m.s deviations**						
Bond lengths (Å)	0.01	0.005	0.005	0.004	0.005	0.005
Bond angles (°)	1.016	0.739	0.709	0.723	0.729	0.801
*The value for the highest-resolution shell is shown in parentheses						

**Variation (4X6 Scramble)**	**J16**	**J19**	**J21**	**J22**	**J23**	**J24**
PDB Code: Junction	7JJ3	7JJ2	7JIQ	7JIP	7JIO	7JIN
PDB Code: Duplex	7JLA	7JLB	7JLC	7JLD	7JLE	7JLF
**Data Collection**						
Beamline	BNL AMX	ALS 5.0.2	APS 19-ID	ALS 5.0.2	APS 19-ID	APS 19-ID
Resolution (Å)	3	3	3.05	3.15	3	2.9
Space group	R3	R3	R3	R3	R3	R3
**Cell dimensions**						
a, b, c (Å)	113.31,133.31,52.18	111.97,111.97,50.99	113.38,113.38,51.31	113.02,113.02,49.34	114.26,114.26,51.47	112.59,112.59,51.86
α, β, γ (°)	90,90,120	90,90,120	90,90,120	90,90,120	90,90,120	90,90,120
Space group	H3	H3	H3	H3	H3	H3
**Cell dimensions**						
a, b, c (Å)	59.48	67.61	67.63	66.87	68.34	65.86
α, β, γ (°)	111.95	113.81	113.84	114.16	113.93	113.7
Wavelength (Å)	1	1	0.92	1	1	1
Total observations	48142	44318	47219	33416	50842	53206
Unique reflections	4978	4591	4622	3586	4894	5331
R_{pim}	2.9 (23.6)	2.4 (21.7)	2.5 (23.7)	2.5 (11.0)	3.4 (22.5)	5.4 (52.2)
$CC_{1/2}$	1.015 (0.825)	0.979 (0.900)	0.943 (0.933)	0.976 (0.974)	1.003 (0.92)	1.064 (0.671)
$I/\sigma I$	33.71 (1.6)	41.57 (1.6)	47.89 (1.57)	35.41 (2.96)	47 (2.71)	49.12 (1.13)
Completeness (%)	99.5 (92.8)	96.8 (78.8)	99.2 (94.7)	89 (52)	99.7 (100)	99.0(96.4)
Redundancy	9.7 (7.5)	9.7 (7.8)	10.2 (8.2)	9.3 (6.3)	10.4 (9.3)	10.0 (7.6)
**Refinement: Junction**						
R_{work}/R_{free}	19.98/22.59	22.79/26.80	18.97/20.93	21.40/23.61	18.68/22.49	22.13/24.17
**No. atoms**						
DNA	854	855	855	855	855	855
ligand/ion	5	2	0	1	4	1
**R.m.s deviations**						
Bond lengths (Å)	0.007	0.004	0.01	0.005	0.01	0.006
Bond angles (°)	0.905	0.657	10639	0.658	1.836	0.778
**Refinement: Duplex**						
R_{work}/R_{free}	21.80/25.36	23.27/27.56	20.66/23.58	19.99/22.67	20.37/22.83	22.49/23.2
**No. atoms**						
DNA	855	855	855	855	855	855
ligand/ion	2	2	0	2	3	2
**R.m.s deviations**						
Bond lengths (Å)	0.005	0.005	0.01	0.006	0.008	0.006
Bond angles (°)	0.737	0.803	1.704	0.806	0.881	0.825

*The value for the highest-resolution shell is shown in parentheses

**Variation (4X6 Scramble)**	**J26**	**J30**	**J31**	**J33**	**J34**	**J36**
PDB Code: Junction	7JIM	7JI9	7JI8	7JI7	7JI6	7JI5
PDB Code: Duplex	7JNJ	7JSB	7JSC	7JNK	7JNL	7JLM
**Data Collection**						
Beamline	BNL FMX	APS 19-ID	APS 19-ID	ALS 5.0.2	ALS 5.0.2	APS 19-ID
Resolution (Å)	3	3.1	2.95	3.1	3	2.7
Space group	R3	R3	R3	R3	R3	R3
**Cell dimensions**						
a, b, c (Å)	112.09,112.09,51.13	114.70.114.70,50.46	113.11,113.11,50.39	113.57,113.57,51.80	111.80.111.80,51.30	112.71,112.71,50.62
α, β, γ (°)	90,90,120	90,90,120	90,90,120	90,90,120	90,90,120	90,90,120
Space group	H3	H3	H3	H3	H3	H3
**Cell dimensions**						
a, b, c (Å)	67.08	69.2	67.39	67.67	66.5	67.14
α, β, γ (°)	113.8	114.34	114	113.78	113.68	113.91
Wavelength (Å)	1	1	0.92	1	1	0.92
Total observations	37394	41400	50184	43588	44343	67034
Unique reflections	4461	4290	4983	4441	4591	6544
R_{pim}	5.3 (47.5)	5.3 (22.0)	2.9 (39.2)	3.9 (19.1)	2.4 (19.5)	2.5 (35.4)
$CC_{1/2}$	0.931 (0.525)	0.955 (0.916)	1.018 (0.786)	0.919 (0.936)	1.05 (0.947)	0.924 (0.746)
$I/\sigma I$	15.5 (0.5)	43.42 (2.22)	52.72 (1.24)	25.6 (1.57)	50.73 (2.03)	54.97 (1.39)
Completeness (%)	94.9 (64.2)	95.8 (83.9)	98.5 (87.6)	98.7 (85.6)	96.3 (70.3)	99.7 (98.8)
Redundancy	8.4 (5.3)	9.7 (6.7)	10.1 (7.3)	9.8 (6.9)	9.7 (8.4)	10.2 (8.8)
**Refinement: Junction**						
R_{work}/R_{free}	22.59/26.13	20.20/22.75	25.46/28.14	21.84/23.14	20.81/24.16	22.91/26.77
**No. atoms**						
DNA	855	855	855	855	855	855
ligand/ion	2	1	1	2	1	1
**R.m.s deviations**						
Bond lengths (Å)	0.005	0.006	0.005	0.005	0.007	0.006
Bond angles (°)	0.68	0.727	0.731	0.769	0.882	0.781
**Refinement: Duplex**						
R_{work}/R_{free}	20.82/24.27	22.72/24.15	25.05/28.22	23.47/24.44	23.23/28.00	22.36/24.35
**No. atoms**						
DNA	855	858	858	858	858	858
ligand/ion	5	1	1	2	4	2
**R.m.s deviations**						
Bond lengths (Å)	0.008	0.005	0.007	0.006	0.007	0.006
Bond angles (°)	0.822	0.748	0.811	0.676	0.764	0.737
*The value for the highest-resolution shell is shown in parentheses						

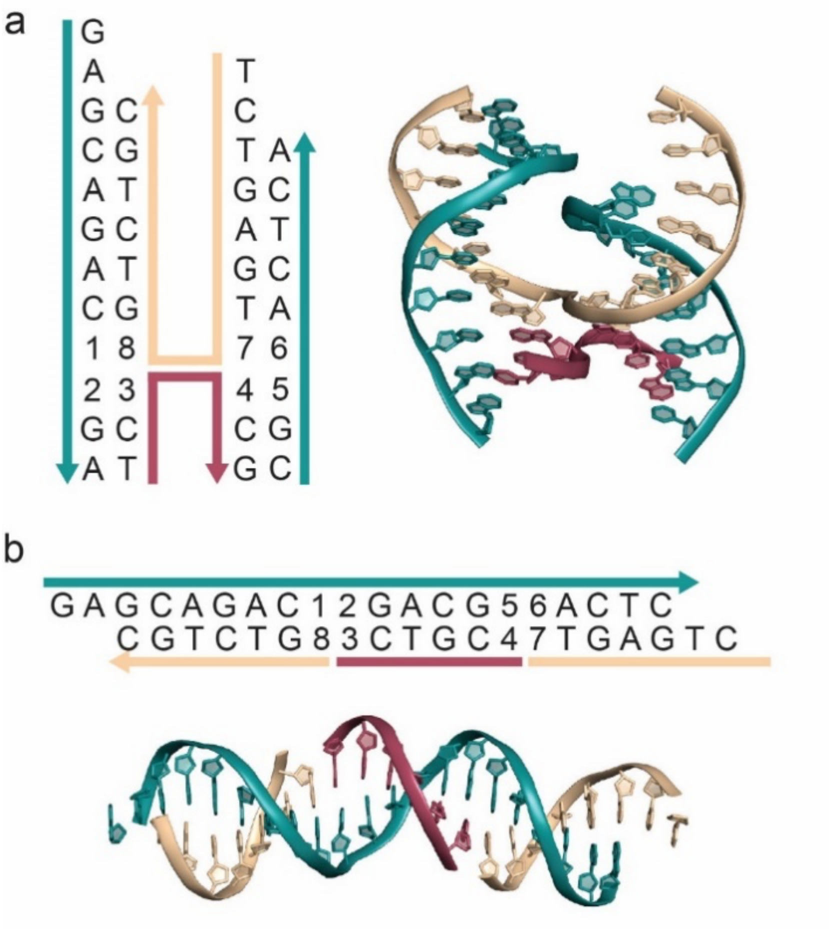

Supplementary Figure 2. Structural representations of junction and duplex models. a) 2D topology and a representative 3D model of a Holliday junction containing the sequence from the 4x6 motif and the specified numbering system used for the assignment of sequence labeled 1-8. b) 2D topology and a representative 3D model when a duplex is represented as the contents of the asymmetric unit with the corresponding junction sequences sequence labeled 1-8. Each of the three component strands are represented as follows: (S1) "the central weaving strand" (red), (S2) which is linear and does not participate in any of the junction crossovers (teal), and (S3) the second crossover strand which forms complementary pairs with S2 on each arm (tan).

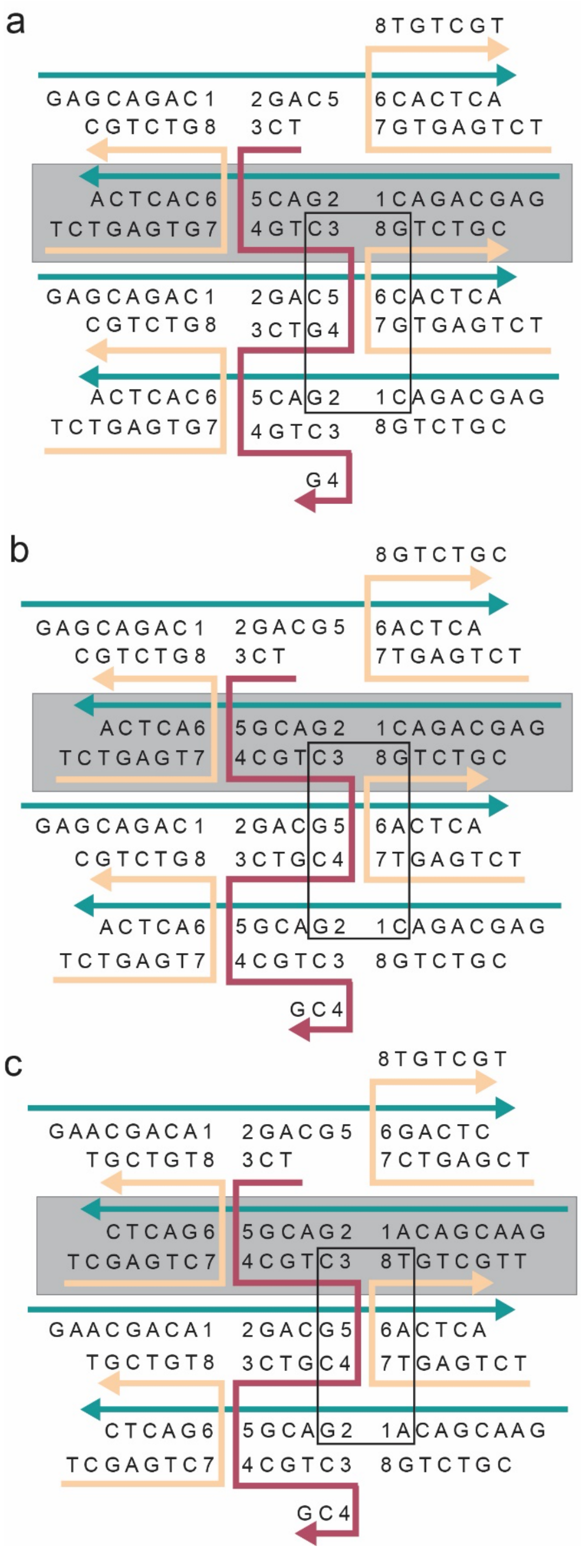

Supplementary Figure 3. 2D topologies of the central building blocks containing Holliday junctions used for the bases of each systems (a) 4x5, (b) 4x6, and (c) 4x6 scramble. 1 of the 4 ASUs (21 bp duplex) that make up the building block is highlighted in gray along with a representative junction that allows the duplexes to assemble in 3D space being boxed.

Supplementary Table 2. DNA sequences used for each constituent oligonucleotide combination for all 36 immobile junctions in each of the three systems used in this work.

Junction	Strand	4x6	4x6 scramble	4x5
**J1**	S1	TCACCGTCACCGTCACCGTCACCG	TCACCGTCACCGTCACCGTCACCG	TCAACGTCACGTCACGTCACG
	S2	GAGCAGACCTGACGGAACTCA	GAACGACACTGACGGAGACTC	GAGCAGACCTGACGACACTCA
	S3	TCTGAGTTGGTCTGC	TCGAGTCTGTGTCGT	TCTGAGTGTGGTCTGC
**J2**	S1	TCATCGTCATCGTCATCGTCATCG	TCATCGTCATCGTCATCGTCATCG	TCAATGTCATGTCATGTCATG
	S2	GAGCAGACCTGACGAGACTCA	GAACGACACTGACGAGGACTC	GAGCAGACCTGACAGCACTCA
	S3	TCTGAGTCGGTCTGC	TCGAGTCCGTGTCGT	TCTGAGTGCGGTCTGC
**J3**	S1	TCAACGTCAACGTCAACGTCAACG	TCAACGTCAACGTCAACGTCAACG	TCAAGTCAAGTCAAGTCAAG
	S2	GAGCAGACGTGACGTCACTCA	GAACGACAGTGACGTCGACTC	GAGCAGACGTGACTCCACTCA
	S3	TCTGAGTGCGTCTGC	TCGAGTCGCTGTCGT	TCTGAGTGGCGTCTGC
**J4**	S1	TCTCCGTCTCCGTCTCCGTCTCCG	TCTCCGTCTCCGTCTCCGTCTCCG	TCTCGTCTCGTCTCGTCTCG
	S2	GAGCAGACCAGACGGGACTCA	GAACGACACAGACGGGGACTC	GAGCAGACCAGACGGCACTCA
	S3	TCTGAGTCGGTCTGC	TCGAGTCCGTGTCGT	TCTGAGTGCGGTCTGC
**J5**	S1	TCGCCGTCGCCGTCGCCGTCGCCG	TCGCCGTCGCCGTCGCCGTCGCCG	TCGCGTCGCGTCGCGTCGCG
	S2	GAGCAGACCCGACGGGACTCA	GAACGACACCGACGGGGACTC	GAGCAGACCCGACGGCACTCA
	S3	TCTGAGTCGGTCTGC	TCGAGTCCGTGTCGT	TCTGAGTGCGGTCTGC
**J6**	S1	TCTACGTCTACGTCTACGTCTACG	TCTACGTCTACGTCTACGTCTACG	TCTAGTCTAGTCTAGTCTAG
	S2	GAGCAGACAAGACGTTACTCA	GAACGACAAAGACGTTGACTC	GAGCAGACAAGACTTCACTCA
	S3	TCTGAGTATGTCTGC	TCGAGTCATTGTCGT	TCTGAGTGATGTCTGC
**J7**	S1	TCTCCGTCTCCGTCTCCGTCTCCG	TCTCCGTCTCCGTCTCCGTCTCCG	TCTCGTCTCGTCTCGTCTCG
	S2	GAGCAGACCAGACGGTACTCA	GAACGACACAGACGGTGACTC	GAGCAGACCAGACGTCACTCA
	S3	TCTGAGTAGGTCTGC	TCGAGTCAGTGTCGT	TCTGAGTGAGGTCTGC
**J8**	S1	TCTTCGTCTTCGTCTTCGTCTTCG	TCTTCGTCTTCGTCTTCGTCTTCG	TCTTGTCTTGTCTTGTCTTG
	S2	GAGCAGACCAGACGACACTCA	GAACGACACAGACGACGACTC	GAGCAGACCAGACACCACTCA
	S3	TCTGAGTGGGTCTGC	TCGAGTCGGTGTCGT	TCTGAGTGGGGTCTGC
**J9**	S1	TCAACGTCAACGTCAACGTCAACG	TCAACGTCAACGTCAACGTCAACG	TCAAGTCAAGTCAAGTCAAG
	S2	GAGCAGACGTGACGTGACTCA	GAACGACAGTGACGTGGACTC	GAGCAGACGTGACTGCACTCA
	S3	TCTGAGTCCGTCTGC	TCGAGTCCCTGTCGT	TCTGAGTGCCGTCTGC
**J10**	S1	TCAACGTCAACGTCAACGTCAACG	TCAACGTCAACGTCAACGTCAACG	TCAAGTCAAGTCAAGTCAAG
	S2	GAGCAGACCTGACGTCACTCA	GAACGACACTGACGTCGACTC	GAGCAGACCTGACTCCACTCA
	S3	TCTGAGTGGGTCTGC	TCGAGTCGGTGTCGT	TCTGAGTGGGGTCTGC
**J11**	S1	TCTTCGTCTTCGTCTTCGTCTTCG	TCTTCGTCTTCGTCTTCGTCTTCG	TCTTGTCTTGTCTTGTCTTG
	S2	GAGCAGACGAGACGAGACTCA	GAACGACAGAGACGAGGACTC	GAGCAGACGAGACAGCACTCA
	S3	TCTGAGTCCGTCTGC	TCGAGTCCCTGTCGT	TCTGAGTGCCGTCTGC
**J12**	S1	TCTTCGTCTTCGTCTTCGTCTTCG	TCTTCGTCTTCGTCTTCGTCTTCG	TCTTGTCTTGTCTTGTCTTG
	S2	GAGCAGACCAGACGAGACTCA	GAACGACACAGACGAGGACTC	GAGCAGACCAGACAGCACTCA
	S3	TCTGAGTCGGTCTGC	TCGAGTCCGTGTCGT	TCTGAGTGCGGTCTGC

Junction	Strand	4x6	4x6 scramble	4x5
**J13**	S1	TCTACGTCTACGTCTACGTCTACG	TCTACGTCTACGTCTACGTCTACG	TCTAGTCTAGTCTAGTCTAG
	S2	GAGCAGACCAGACGAGACTCA	GAACGACACAGACGTGGACTC	GAGCAGACCAGACTGCACTCA
	S3	TCTGAGTCGGTCTGC	TCGAGTCCGTGTCGT	TCTGAGTGCGGTCTGC
**J14**	S1	TCTGCGTCTGCGTCTGCGTCTGCG	TCTGCGTCTGCGTCTGCGTCTGCG	TCTGGTCTGGTCTGGTCTGG
	S2	GAGCAGACGAGACGCCACTCA	GAACGACAGAGACGCCGACTC	GAGCAGACGAGACCCCACTCA
	S3	TCTGAGTGCGTCTGC	TCGAGTCGCTGTCGT	TCTGAGTGGCGTCTGC
**J15**	S1	TCAGCGTCAGCGTCAGCGTCAGCG	TCAGCGTCAGCGTCAGCGTCAGCG	TCAGGTCAGGTCAGGTCAGG
	S2	GAGCAGACGTGACGCGACTCA	GAACGACAGTGACGCGGACTC	GAGCAGACGTGACCGCACTCA
	S3	TCTGAGTCCGTCTGC	TCGAGTCCCTGTCGT	TCTGAGTGCCGTCTGC
**J16**	S1	TCAGCGTCAGCGTCAGCGTCAGCG	TCAGCGTCAGCGTCAGCGTCAGCG	TCAGGTCAGGTCAGGTCAGG
	S2	GAGCAGACGTGACGCCACTCA	GAACGACAGTGACGCCGACTC	GAGCAGACGTGACCCCACTCA
	S3	TCTGAGTGCGTCTGC	TCGAGTCGCTGTCGT	TCTGAGTGGCGTCTGC
**J17**	S1	TCTACGTCTACGTCTACGTCTACG	TCTACGTCTACGTCTACGTCTACG	TCTAGTCTAGTCTAGTCTAG
	S2	GAGCAGACGAGACGTGACTCA	GAACGACAGAGACGTGGACTC	GAGCAGACGAGACTGCACTCA
	S3	TCTGAGTCCGTCTGC	TCGAGTCCCTGTCGT	TCTGAGTGCCGTCTGC
**J18**	S1	TCTTCGTCTTCGTCTTCGTCTTCG	TCTTCGTCTTCGTCTTCGTCTTCG	TCTTGTCTTGTCTTGTCTTG
	S2	GAGCAGACGAGACGACACTCA	GAACGACAGAGACGACGACTC	GAGCAGACGAGACACCACTCA
	S3	TCTGAGTGCGTCTGC	TCGAGTCGCTGTCGT	TCTGAGTGGCGTCTGC
**J19**	S1	TCTACGTCTACGTCTACGTCTACG	TCTACGTCTACGTCTACGTCTACG	TCTAGTCTAGTCTAGTCTAG
	S2	GAGCAGACGAGACGTCACTCA	GAACGACAGAGACGTCGACTC	GAGCAGACGAGACTCCACTCA
	S3	TCTGAGTGCGTCTGC	TCGAGTCGCTGTCGT	TCTGAGTGGCGTCTGC
**J20**	S1	TCATCGTCATCGTCATCGTCATCG	TCATCGTCATCGTCATCGTCATCG	TCATGTCATGTCATGTCATG
	S2	GAGCAGACGTGACGAGACTCA	GAACGACAGTGACGAGGACTC	GAGCAGACGTGACAGCACTCA
	S3	TCTGAGTCGGTCTGC	TCGAGTCCGTGTCGT	TCTGAGTGCCGTCTGC
**J21**	S1	TCAACGTCAACGTCAACGTCAACG	TCAACGTCAACGTCAACGTCAACG	TCAAGTCAAGTCAAGTCAAG
	S2	GAGCAGACCTGACGTGACTCA	GAACGACACTGACGTGGACTC	GAGCAGACCTGACTGCACTCA
	S3	TCTGAGTCGGTCTGC	TCGAGTCCGTGTCGT	TCTGAGTGCGGTCTGC
**J22**	S1	TCACCGTCACCGTCACCGTCACCG	TCACCGTCACCGTCACCGTCACCG	TCACGTCACGTCACGTCACG
	S2	GAGCAGACCTGACGGCACTCA	GAACGACACTGACGGCGACTC	GAGCAGACCTGACGCCACTCA
	S3	TCTGAGTGGGTCTGC	TCGAGTCGGTGTCGT	TCTGAGTGGGGTCTGC
**J23**	S1	TCATCGTCATCGTCATCGTCATCG	TCATCGTCATCGTCATCGTCATCG	TCATGTCATGTCATGTCATG
	S2	GAGCAGACCTGACGACACTCA	GAACGACACTGACGACGACTC	GAGCAGACCTGACACCACTCA
	S3	TCTGAGTGGGTCTGC	TCGAGTCGGTGTCGT	TCTGAGTGGGGTCTGC
**J24**	S1	TCTACGTCTACGTCTACGTCTACG	TCTACGTCTACGTCTACGTCTACG	TCTAGTCTAGTCTAGTCTAG
	S2	GAGCAGACCAGACGTCACTCA	GAACGACACAGACGTCGACTC	GAGCAGACCAGACTCCACTCA
	S3	TCTGAGTGGGTCTGC	TCGAGTCGGTGTCGT	TCTGAGTGGGGTCTGC

Junction	Strand	4x6	4x6 scramble	4x5
**J25**	S1	TCCGCGTCCGCGTCCGCGTCCGCG	TCCGCGTCCGCGTCCGCGTCCGCG	TCTGGTCTGGTCTGGTCTGG
	S2	GAGCAGACGAGACGTGACTCA	GAACGACAGAGACGTCGACTC	GAGCAGACGAGACCGCACTCA
	S3	TCTGAGTCCGTCTGC	TCGAGTCCCTGTCGT	TCTGAGTGCCGTCTGC
**J26**	S1	TCATCGTCATCGTCATCGTCATCG	TCATCGTCATCGTCATCGTCATCG	TCATGTCATGTCATGTCATG
	S2	GAGCAGACTTGACGACACTCA	GAACGACATTGACGACGACTC	GAGCAGACTTGACACCACTCA
	S3	TCTGAGTAGGTCTGC	TCATCGTCATCGTCATCGTCATCG	TCTGAGTGAGGTCTGC
**J27**	S1	TCTTCGTCTTCGTCTTCGTCTTCG	TCTTCGTCTTCGTCTTCGTCTTCG	TCTTGTCTTGTCTTGTCTTG
	S2	GAGCAGACTAGACGAGACTCA	GAACGACATAGACGAGGACTC	GAGCAGACTAGACAGCACTCA
	S3	TCTGAGTCAGTCTGC	TCGAGTCCATGTCGT	TCTGAGTGCAGTCTGC
**J28**	S1	TCAACGTCAACGTCAACGTCAACG	TCAACGTCAACGTCAACGTCAACG	TCAAGTCAAGTCAAGTCAAG
	S2	GAGCAGACATGACGTCACTCA	GAACGACAATGACGTCGACTC	GAGCAGACATGACTCCACTCA
	S3	TCTGAGTGTGTCTGC	TCGAGTCGTTGTCGT	TCTGAGTGGTGTCTGC
**J29**	S1	TCTACGTCTACGTCTACGTCTACG	TCTACGTCTACGTCTACGTCTACG	TCTAGTCTAGTCTAGTCTAG
	S2	GAGCAGACAAGACGTGACTCA	GAACGACAAAGACGTGGACTC	GAGCAGACAAGACTGCACTCA
	S3	TCTGAGTCTGTCTGC	TCGAGTCCTTGTCGT	TCTGAGTGCTGTCTGC
**J30**	S1	TCACCGTCACCGTCACCGTCACCG	TCACCGTCACCGTCACCGTCACCG	TCACGTCACGTCACGTCACG
	S2	GAGCAGACCTGACGGGACTCA	GAACGACACTGACGGGGACTC	GAGCAGACCTGACGGCACTCA
	S3	TCTGAGTCGGTCTGC	TCGAGTCCGTGTCGT	TCTGAGTGCGGTCTGC
**J31**	S1	TCTCCGTCTCCGTCTCCGTCTCCG	TCTCCGTCTCCGTCTCCGTCTCCG	TCTCGTCTCGTCTCGTCTCG
	S2	GAGCAGACCAGACGGCACTCA	GAACGACACAGACGGCGACTC	GAGCAGACCAGACGCCACTCA
	S3	TCTGAGTGGGTCTGC	TCGAGTCGGTGTCGT	TCTGAGTGGGGTCTGC
**J32**	S1	TCTTCGTCTTCGTCTTCGTCTTCG	TCTTCGTCTTCGTCTTCGTCTTCG	TCTTGTCTTGTCTTGTCTTG
	S2	GAGCAGACTAGACGACACTCA	GAACGACATAGACGACGACTC	GAGCAGACTAGACACCACTCA
	S3	TCTGAGTGAGTCTGC	TCGAGTCGATGTCGT	TCTGAGTGGAGTCTGC
**J33**	S1	TCATCGTCATCGTCATCGTCATCG	TCATCGTCATCGTCATCGTCATCG	TCATGTCATGTCATGTCATG
	S2	GAGCAGACTTGACGAGACTCA	GAACGACATTGACGAGGACTC	GAGCAGACTTGACAGCACTCA
	S3	TCTGAGTCAGTCTGC	TCGAGTCCATGTCGT	TCTGAGTGCAGTCTGC
**J34**	S1	TCTACGTCTACGTCTACGTCTACG	TCTACGTCTACGTCTACGTCTACG	TCTAGTCTAGTCTAGTCTAG
	S2	GAGCAGACAAGACGTCACTCA	GAACGACAAAGACGTCGACTC	GAGCAGACAAGACTCCACTCA
	S3	TCTGAGTGTGTCTGC	TCGAGTCGTTGTCGT	TCTGAGTGGTGTCTGC
**J35**	S1	TCAACGTCAACGTCAACGTCAACG	TCAACGTCAACGTCAACGTCAACG	TCAAGTCAAGTCAAGTCAAG
	S2	GAGCAGACATGACGTGACTCA	GAACGACAATGACGTGGACTC	GAGCAGACATGACTGCACTCA
	S3	TCTGAGTCTGTCTGC	TCGAGTCCTTGTCGT	TCTGAGTGCTGTCTGC
**J36**	S1	TCATCGTCATCGTCATCGTCATCG	TCATCGTCATCGTCATCGTCATCG	TCATGTCATGTCATGTCATG
	S2	GAGCAGACGTGACGACACTCA	GAACGACAGTGACGACGACTC	GAGCAGACGTGACACCACTCA
	S3	TCTGAGTGCGTCTGC	TCGAGTCGCTGTCGT	TCTGAGTGGCGTCTGC

Supplementary Table 3. Components for each of the 48 buffer conditions used for sparse matrix screening as the starting point for each crystallization setup. After determination of conditions whereby successful crystals were or were not acquired, rigorous fine grid screening was performed by modification of conditions such as DNA concentration, salt or solvent concentration, pH, or surfactant concentration to either optimize or promote crystallization in order to determine which junctions were either viable or "fatal". The original screen was adapted from a discontinued Sigma-Aldrich product, which can be found at https://www.sigmaaldrich.com/catalog/product/sigma/80701?lang=en&region=US.

**1**	0.05 M HEPES pH 7.5	80 mM $MgCl_2$	2.5 mM spermine			
**2**	0.05 M Na cacodylate pH 6.0	18 mM $MgCl_2$	2.25 mM spermine	1 mM $CuSO_4$	9% isopropanol	
**3**	0.05 M Na cacodylate pH 6.5	18 mM $MgCl_2$	0.9 mM spermine	1.8 mM $CoH_{18}N_6$	9% isopropanol	
**4**	0.05 M Na cacodylate pH 6.5	18 mM $MgCl_2$	2.25 mM spermine	9% isopropanol		
**5**	0.05 M Na cacodylate pH 7.0	18 mM $MgCl_2$	2.25 mM spermine	0.9 mM $CoH_{18}N_6$	4.5% MPD	
**6**	0.05 M Na cacodylate pH 6.5	36 mM $MgCl_2$	2.25 mM spermine	5% PEG 400		
**7**	0.05 M Na succinate pH 5.5	10 mM $MgCl_2$	2.0 mM $CoH_{18}N_6$	10% isopropanol		
**8**	0.05 M Na cacodylate pH 6.0	20 mM $MgCl_2$	1.0 mM spermine	15% ethanol		
**9**	0.05 M Na cacodylate pH 7.0	20 mM $MgCl_2$	1.0 mM spermine	1.0 mM $CoH_{18}N_6$	15% ethanol	
**10**	0.05 M Na cacodylate pH 7.0	5 mM $MgCl_2$	1.0 mM spermine	10% tert-butanol		
**11**	0.05 M Na cacodylate pH 7.0	30 mM $MgCl_2$	2.5 mM spermine	5% PEG 400		
**12**	0.05 M Na cacodylate pH 6.5	100 mM $MgCl_2$	2.0 mM $CoH_{18}N_6$	5% isopropanol		
**13**	0.05 M Tris pH 8.0	10 mM $MgCl_2$	1.0 mM $CoH_{18}N_6$	20% ethanol		
**14**	0.05 M HEPES pH 7.5	20 mM $MgCl_2$	1.0 mM spermine	5% PEG 8000		
**15**	0.05 M Na cacodylate pH 6.0	20 mM $MgCl_2$	2.5 mM spermine	5% PEG 4000		
**16**	0.05 M Na cacodylate pH 6.0	10 mM $MgCl_2$	2.5 mM spermine	5 mM $CaCl_2$	10% isopropanol	
**17**	0.05 M Na cacodylate pH 7.0	9 mM $MgCl_2$	2.25 mM spermine	1.8 mM $CoH_{18}N_6$	0.9 mM spermidine	5% PEG 400
**18**	0.05 M Na cacodylate pH 6.5	10 mM $MgCl_2$	2.5 mM spermine	1 mM $CuSO_4$	10% isopropanol	
**19**	0.05 M Na cacodylate pH 6.0	20 mM $MgCl_2$	1.0 mM spermine	2 mM $CaCl_2$	10% MPD	
**20**	0.05 M HEPES pH 7.5	15 mM $MgCl_2$	1.0 mM spermidine	10% dioxan		
**21**	0.05 M Na cacodylate pH 6.0	15 mM $MgCl_2$	3.0 mM spermine	10% PEG 400		
**22**	0.05 M Na cacodylate pH 6.5	2.5 mM spermine	18 mM $CaCl_2$	9% isopropanol		
**23**	0.05 M Na cacodylate pH 6.5	2.0 mM spermine	1.0 mM $CoH_{18}N_6$	80 mM $CaCl_2$		
**24**	0.05 M Na cacodylate pH 6.5	5 mM $MgCl_2$	2.5 mM $CoH_{18}N_6$			
**25**	0.05 M Na cacodylate pH 6.5	30 mM $MgCl_2$	1.0 mM spermine	1.3 M Li_2SO_4		
**26**	0.05 M Na cacodylate pH 6.0	200 mM $Ca(CH_3COO)_2$	5% isopropanol			
**27**	0.05 M Na cacodylate pH 6.5	100 mM MgCl2	1.0 mM $CoH_{18}N_6$	10% ethanol		
**28**	0.05 M Na cacodylate pH 6.0	10 mM $MgCl_2$	2.5 mM spermidine	2.5 M NaCl		
**29**	0.05 M Na cacodylate pH 6.5	10 mM $MgCl_2$	200 mM sodium citrate	5% isopropanol		
**30**	0.05 M Na cacodylate pH 6.5	15 mM $MgCl_2$	10 mM spermine	2.0 M Li_2SO_4		
**31**	0.05 M Na cacodylate pH 6.5	20 mM $MgCl_2$	1.0 mM spermine	2.0 M $(NH_4)_2SO_4$		
**32**	0.05 M Na cacodylate pH 6.5	10 mM $MgCl_2$	1.5 mM spermine	3.0 M $(NH_4)_2SO_4$		
**33**	0.05 M HEPES pH 7.5	15 mM $MgCl_2$	1.0 mM spermine	1.0 M $(NH_4)_2SO_4$		
**34**	0.05 M Na cacodylate pH 6.0	200 mM $Ca(CH_3COO)_2$	2.5 M NaCl			
**35**	0.05 M Na cacodylate pH 6.0	200 mM $Ca(CH_3COO)_2$	1.0 mM $CoH_{18}N_6$	2.0 M LiCl		
**36**	0.05 M Na cacodylate pH 6.5	15 mM $MgCl_2$	5.0 mM spermidine	1.0 mM $CoH_{18}N_6$	2.0 M NaCl	
**37**	0.05 M Na cacodylate pH 6.5	200 mM $MgCl_2$	100 mM NaCl	20% PEG 1000		
**38**	0.05 M Tris pH 7.5	50 mM $MgCl_2$	1.0 M sodium tartarate			
**39**	0.05 M Tris pH 7.5	200 mM $MgCl_2$	2.5 M NaCl			
**40**	0.05 M Na cacodylate pH 6.0	200 mM $MgCl_2$	2.5 M KCl			
**41**	0.05 M Tris pH 8.0	200 mM $MgCl_2$	15% ethanol			
**42**	0.05 M Na cacodylate pH 6.0	15 mM $MgCl_2$	5.0 mM spermidine	2.0 M Li_2SO_4		
**43**	0.05 M Na cacodylate pH 6.0	20 mM $Mg(CH_3COO)_2$	0.5 mM spermine	100 mM NaCl	25% MPD	
**44**	0.05 M Na succinate pH 5.5	20 mM $MgCl_2$	0.5 mM spermine	3.0 M $(NH_4)_2SO_4$		
**45**	0.05 M Na cacodylate pH 6.5	5.0 mM $CoH_{18}N_6$	2.5 M KCl			
**46**	0.05 M Na cacodylate pH 6.5	50 mM $MgCl_2$	2.0 mM $CoH_{18}N_6$	1.5 M Li_2SO_4		
**47**	0.05 M Na cacodylate pH 6.5	1.0 mM spermine	2.0 mM $CoH_{18}N_6$	30 mM $CaCl_2$	2.0 M LiCl	
**48**	0.05 M Na cacodylate pH 6.5	10 mM $MgCl_2$	50 mM spermine			

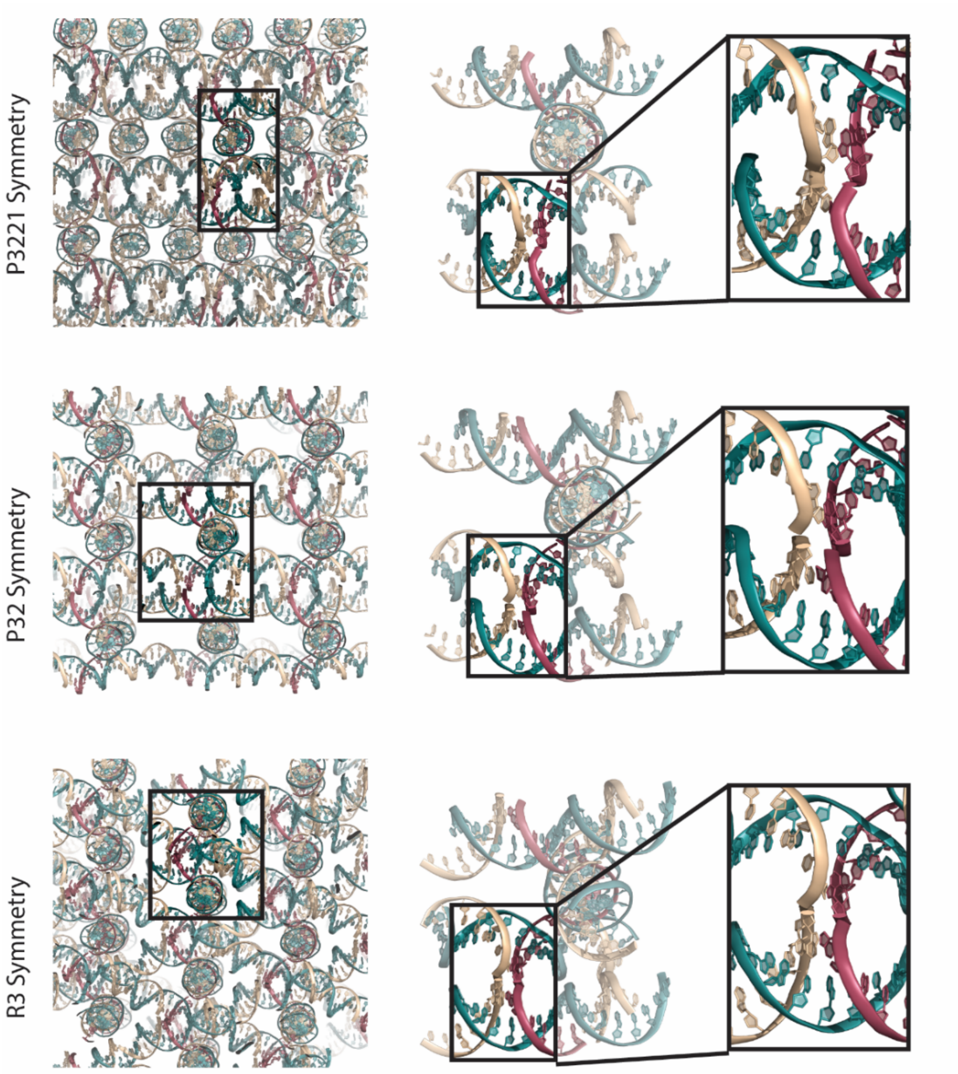

Supplementary Figure 4. Observed lattice packing from three unique space groups. Crystallographic symmetries exhibited by the 4x5 and 4x6 motifs with various immobile junctions. The Holliday junction (right) is the central component of the structure, and is comprised of two crossover strands: S1 (red) and S3 (tan) and a continuous linear strand that pairs at complementary sequences with the crossover strands to complete each of the double helical arms. The unit required for assembly of the full lattice (left) contains four 21 bp duplexes with a single junction (black box) between each helix, and that are tethered by an oligonucleotide with four repeats of either 5 or 6 bases. Each resulting duplex is tailed by complementary 2 base "sticky ends" which mediate the assembly of the crystal by forming continuous helical arrays from the pairing of each constituent "block" (boxed). Although each systems are closely related, and form using the same design principles, the resulting lattices are strikingly different as a result of discrete angular differences exerted by each junction.

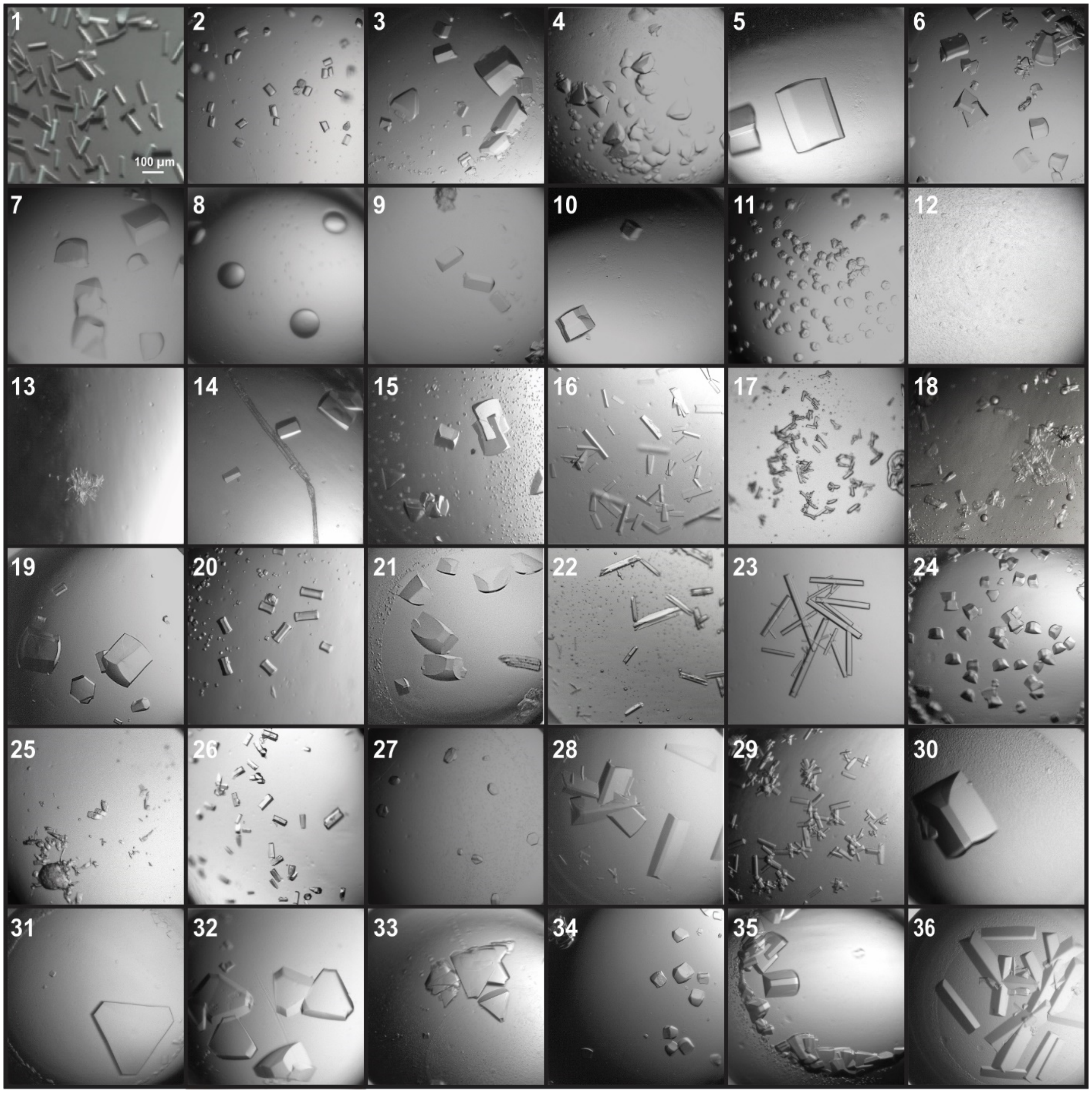

Supplementary Figure 5. Representative bright-field images from the crystallization screen of all 36 immobile Holliday junctions in the 4x5 system. The buffers that correspond to each image are indicated in Supplementary table 3, and the resulting viability of each crystal type used for structure solution, or for those conditions ultimately determined as "fatal", can be found in Supplementary table 4.

Supplementary Table 4. The buffer conditions used for the corresponding bright field images shown in Supplementary figure 6 for the 4x5 system.

Junction	Buffer	Buffer Components					
1	8	0.05 M Na cacodylate pH 6.0	20 mM $MgCl_2$	1.0 mM spermine	15% ethanol		
2	15	0.05 M Na cacodylate pH 6.0	20 mM $MgCl_2$	2.5 mM spermine	5% PEG 4000		
3	1	0.05 M HEPES pH 7.5	80 mM $MgCl_2$	2.5 mM spermine			
4	9	0.05 M Na cacodylate pH 7.0	20 mM $MgCl_2$	1.0 mM spermine	1.0 mM $CoH_{18}N_6$	15% ethanol	
5	23	0.05 M Na cacodylate pH 6.5	2.0 mM spermine	1.0 mM $CoH_{18}N_6$	80 mM $CaCl_2$		
6	1	0.05 M HEPES pH 7.5	80 mM $MgCl_2$	2.5 mM spermine			
7	9	0.05 M Na cacodylate pH 7.0	20 mM $MgCl_2$	1.0 mM spermine	1.0 mM $CoH_{18}N_6$	15% ethanol	
8	8	0.05 M Na cacodylate pH 6.0	20 mM $MgCl_2$	1.0 mM spermine	15% ethanol		
9	11	0.05 M Na cacodylate pH 7.0	30 mM $MgCl_2$	2.5 mM spermine	5% PEG 400		
10	11	0.05 M Na cacodylate pH 7.0	30 mM $MgCl_2$	2.5 mM spermine	5% PEG 400		
11	15	0.05 M Na cacodylate pH 6.0	20 mM $MgCl_2$	2.5 mM spermine	5% PEG 4000		
12	21	0.05 M Na cacodylate pH 6.0	15 mM $MgCl_2$	3.0 mM spermine	10% PEG 400		
13	14	0.05 M HEPES pH 7.5	20 mM $MgCl_2$	1.0 mM spermine	5% PEG 8000		
14	16	0.05 M Na cacodylate pH 6.0	10 mM $MgCl_2$	2.5 mM spermine	5 mM $CaCl_2$	10% isopropanol	
15	5	0.05 M Na cacodylate pH 7.0	18 mM $MgCl_2$	2.25 mM spermine	0.9 mM $CoH_{18}N_6$	4.5% MPD	
16	6	0.05 M Na cacodylate pH 6.5	36 mM $MgCl_2$	2.25 mM spermine	5% PEG 400		
17	17	0.05 M Na cacodylate pH 7.0	9 mM $MgCl_2$	2.25 mM spermine	1.8 mM $CoH_{18}N_6$	0.9 mM spermidine	5% PEG 400
18	15	0.05 M Na cacodylate pH 6.0	20 mM $MgCl_2$	2.5 mM spermine	5% PEG 4000		
19	16	0.05 M Na cacodylate pH 6.0	10 mM $MgCl_2$	2.5 mM spermine	5 mM $CaCl_2$	10% isopropanol	
20	6	0.05 M Na cacodylate pH 6.5	36 mM $MgCl_2$	2.25 mM spermine	5% PEG 400		
21	1	0.05 M HEPES pH 7.5	80 mM $MgCl_2$	2.5 mM spermine			
22	3	0.05 M Na cacodylate pH 6.5	18 mM $MgCl_2$	0.9 mM spermine	1.8 mM $CoH_{18}N_6$	9% isopropanol	
23	6	0.05 M Na cacodylate pH 6.5	36 mM $MgCl_2$	2.25 mM spermine	5% PEG 400		
24	4	0.05 M Na cacodylate pH 6.5	18 mM $MgCl_2$	2.25 mM spermine	9% Isopropanol		
25	13	0.05 M TRIS pH 8.0	10 mM $MgCl_2$	1.0 mM $CoH_{18}N_6$	20% ethanol		
26	5	0.05 M Na cacodylate pH 7.0	18 mM $MgCl_2$	2.25 mM spermine	0.9 mM $CoH_{18}N_6$	4.5% MPD	
27	23	0.05 M Na cacodylate pH 6.5	2.0 mM spermine	1.0 mM $CoH_{18}N_6$	80 mM $CaCl_2$		
28	6	0.05 M Na cacodylate pH 6.5	36 mM $MgCl_2$	2.25 mM spermine	5% PEG 400		
29	14	0.05 M HEPES pH 7.5	20 mM $MgCl_2$	1.0 mM spermine	5% PEG 8000		
30	8	0.05 M Na cacodylate pH 6.0	20 mM $MgCl_2$	1.0 mM spermine	15% ethanol		
31	17	0.05 M Na cacodylate pH 7.0	9 mM $MgCl_2$	2.25 mM spermine	1.8 mM $CoH_{18}N_6$	0.9 mM spermidine	5% PEG 400
32	17	0.05 M Na cacodylate pH 7.0	9 mM $MgCl_2$	2.25 mM spermine	1.8 mM $CoH_{18}N_6$	0.9 mM spermidine	5% PEG 400
33	11	0.05 M Na cacodylate pH 7.0	30 mM $MgCl_2$	2.5 mM spermine	5% PEG 400		
34	13	0.05 M TRIS pH 8.0	10 mM $MgCl_2$	1.0 mM $CoH_{18}N_6$	20% ethanol		
35	5	0.05 M Na cacodylate pH 7.0	18 mM $MgCl_2$	2.25 mM spermine	0.9 mM $CoH_{18}N_6$	4.5% MPD	
36	9	0.05 M Na cacodylate pH 7.0	20 mM $MgCl_2$	1.0 mM spermine	1.0 mM $CoH_{18}N_6$	15% ethanol	

Supplementary Table 5. Unit cell dimensions for each corresponding 4x5 junction crystal. The structures were divided according to their respective crystal symmetry with the axis lengths indicated. The average and standard deviation for the respective axes of the two symmetries were also calculated and are shown in bold in the final row. All angles for each space group are $\alpha = \beta = 90°$ $\gamma = 120°$.

**4x5 $P3_221$**		
Junction	a,b	c
1	67.9	59.3
5	67.9	59.5
7	67.8	60.5
10	68.8	62
14	68.9	62.1
19	68.5	60.8
20	67.6	60.4
23	68.5	60.2
26	67.6	60.6
**Average**	**68.16±0.48**	**60.60±0.90**

**4x5 $P3_2$**		
Junction	a,b	c
3	68.9	60.7
6	68.9	59.4
8	68.9	59.8
9	68.7	60.8
15	68.8	60.9
16	69	61.3
21	69	59.4
22	69.4	59.4
24	68.5	60.2
25	69	59.5
28	69	60.6
29	68.7	58.1
31	68.9	59
32	69	60.8
33	68.4	61.3
34	68.8	59.7
35	68.7	60.1
36	68.8	60.6
**Average**	**68.86±0.21**	**60.09±0.84**

.

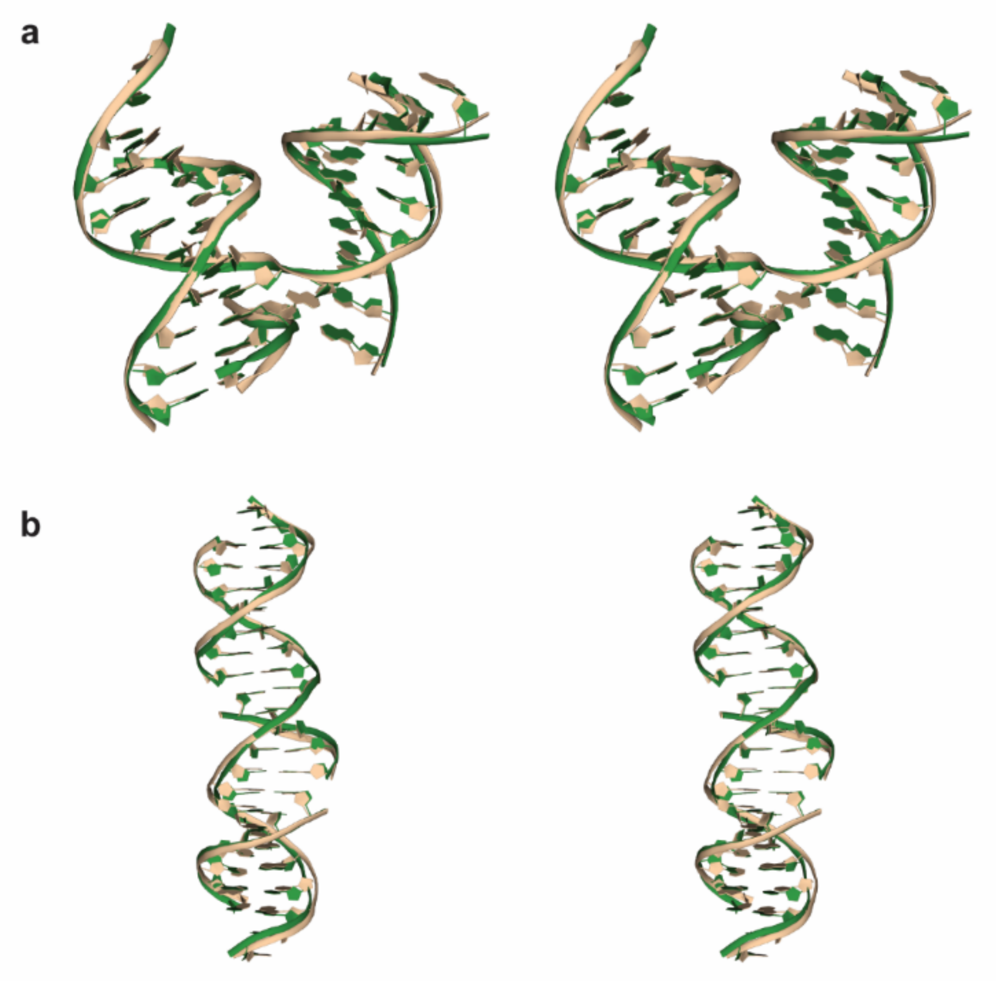

Supplementary Figure 6. Superimposed 4x5 structures of representative *P*$3_2$21 vs. *P*3_2 crystals. (a) and (b) J10 (green) and J9 (tan) are shown (*P*$3_2$21 and *P*3_2, respectively) with their corresponding junction (a) and duplex (b) structures superimposed. The two junction alignments had a global RMSD value of 1.054 and the two duplex alignments had a global RMSD value of 1.207. The differences between the average angles of the *P*$3_2$21 (56.59±1.50) and *P*3_2 (56.05±1.63) are negligible, and no significantly obvious visual differences are apparent; however, the resulting global influence that an even modest difference in angle can have on overall packing is undeniably evident in Supplementary Fig. 8.

Supplementary Table 6. Summary of the resulting space group and corresponding resolution for all 4x5 crystal structures. All 36 of each junction type are shown, with those determined as "fatal" highlighted in red.

Junction	Space Group	Resolution
1	$P3_221$	3.10
2	—	—
3	$P3_2$	3.00
4	—	—
5	$P3_221$	3.15
6	$P3_2$	2.90
7	$P3_221$	3.05
8	$P3_2$	3.10
9	$P3_2$	3.05
10	$P3_221$	3.05
11	—	—
12	—	—
13	—	—
14	$P3_221$	2.85
15	$P3_2$	3.00
16	$P3_2$	3.05
17	—	—
18	—	—

Junction	Space Group	Resolution
19	$P3_221$	2.75
20	$P3_221$	3.10
21	$P3_2$	3.10
22	$P3_2$	3.10
23	$P3_221$	3.05
24	$P3_2$	3.10
25	$P3_2$	3.10
26	$P3_221$	3.10
27	—	—
28	$P3_2$	3.05
29	$P3_2$	3.10
30	—	—
31	$P3_2$	3.15
32	$P3_2$	3.05
33	$P3_2$	3.10
34	$P3_2$	3.00
35	$P3_2$	3.05
36	$P3_2$	3.15

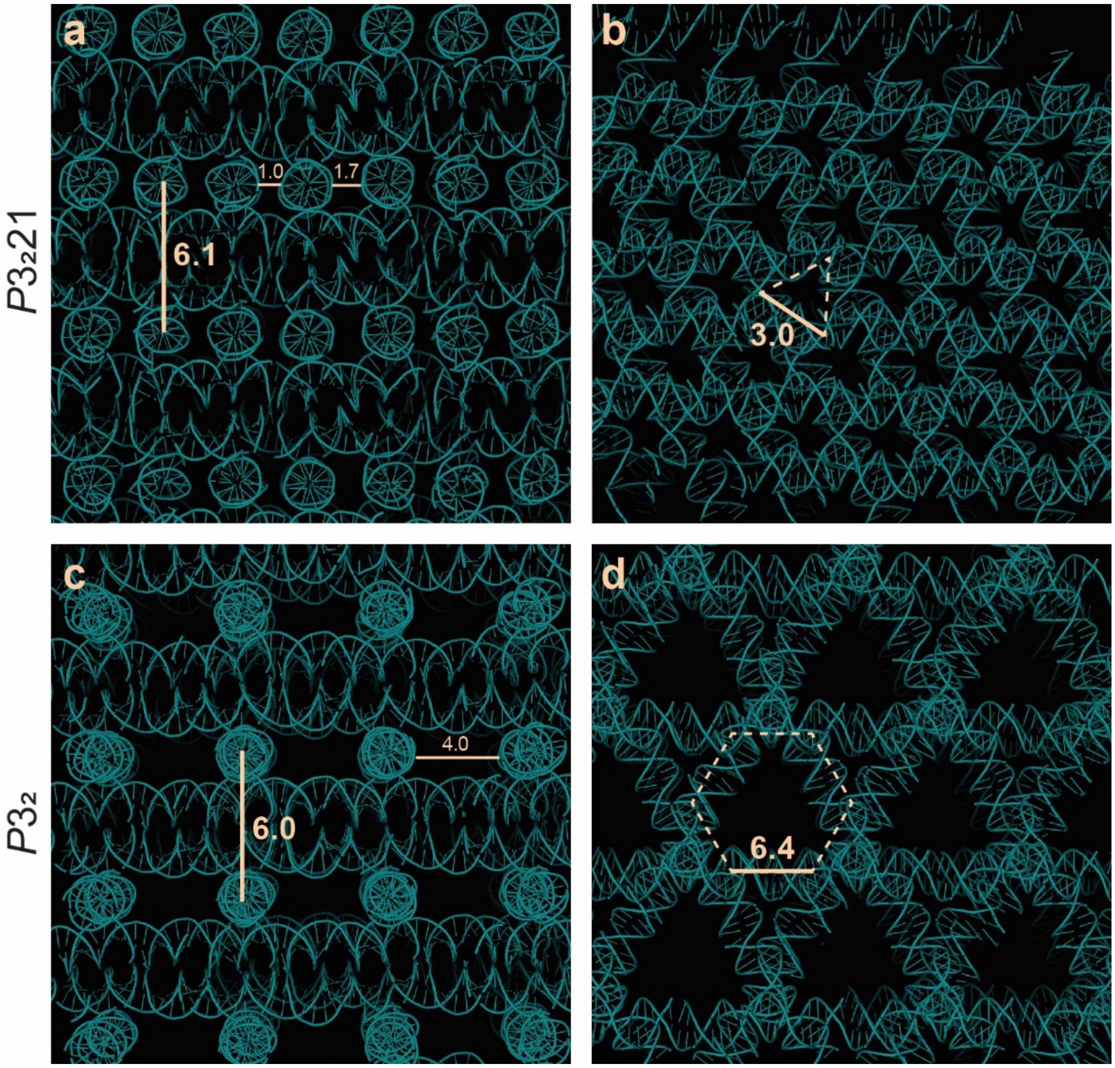

Supplementary Figure 7. Symmetry related duplexes show the full crystal lattice for both symmetries observed in the 4x5 motif. Panels a) and b) correspond to the $P3_221$ symmetric lattices and are rotated 90° with respect to one another. (a) The aperiodicity of the resulting cavities in the 4x5 system with $P3_221$ symmetry are evident in (a) where the corresponding cavity cross-sections are ~1 and 1.7 nm, respectively. The height from the top to the bottom of each 4 layer block is indicated (6.1 nm) and is in good agreement to the average c-axis of the $P3_221$ lattices (60.60 ± 0.90). (b) View along the three-fold crystal axis with an edge length of 3 nm along each edge. The corresponding cavity volumes were calculated as a triangular prism with an edge length of 3.0 nm and a height of 6.1 nm. See main text for additional details. Panels c) and d) correspond to the $P3_2$ lattices and are rotated 90° with respect to one another. (c) The highly periodic array of cavities in the scaffold contain a cross-section length of X, which amounts to a X to Y fold expansion compared to (a). The measured values used to determine the cavity volume, calculated as a hexagonal prism are displayed with an edge length of 6.4 nm and a height of 6.0. The height from the top to the bottom of each 4 layer block is indicated (6.0 nm) and is in good agreement to the average c-axis of the $P3_2$ lattices (60.09 ± 0.84). (d) 90° rotation viewed with six edges ~6.4 nm in length. The corresponding cavity volumes were calculated as a hexagonal prism with an edge length of 6.4 nm and a height of 6.0 nm. See main text for additional details.

Supplementary Table 7. Calculated interduplex angles (IDA) corresponding to each junction structure for the 4x5 system. The listed junctions are divided according to crystal symmetry with each respective angle listed. The average and standard deviation for the respective angles of the two symmetries were also calculated and are shown in bold in the final row.

**4x5**			
***P*3$_2$21**		***P*3$_2$**	
Junction	Angle	Junction	Angle
1	56.84	3	55.20
5	58.18	6	55.12
7	54.69	8	56.81
10	58.56	9	55.94
14	55.32	15	57.81
19	55.19	16	54.53
20	56.04	21	58.44
23	58.51	22	56.46
26	55.98	24	59.52
**56.59±1.50**		25	55.61
		28	53.54
		29	57.36
		31	55.62
		32	55.46
		33	56.88
		34	52.89
		35	55.89
		36	55.77
		**56.05±1.63**	

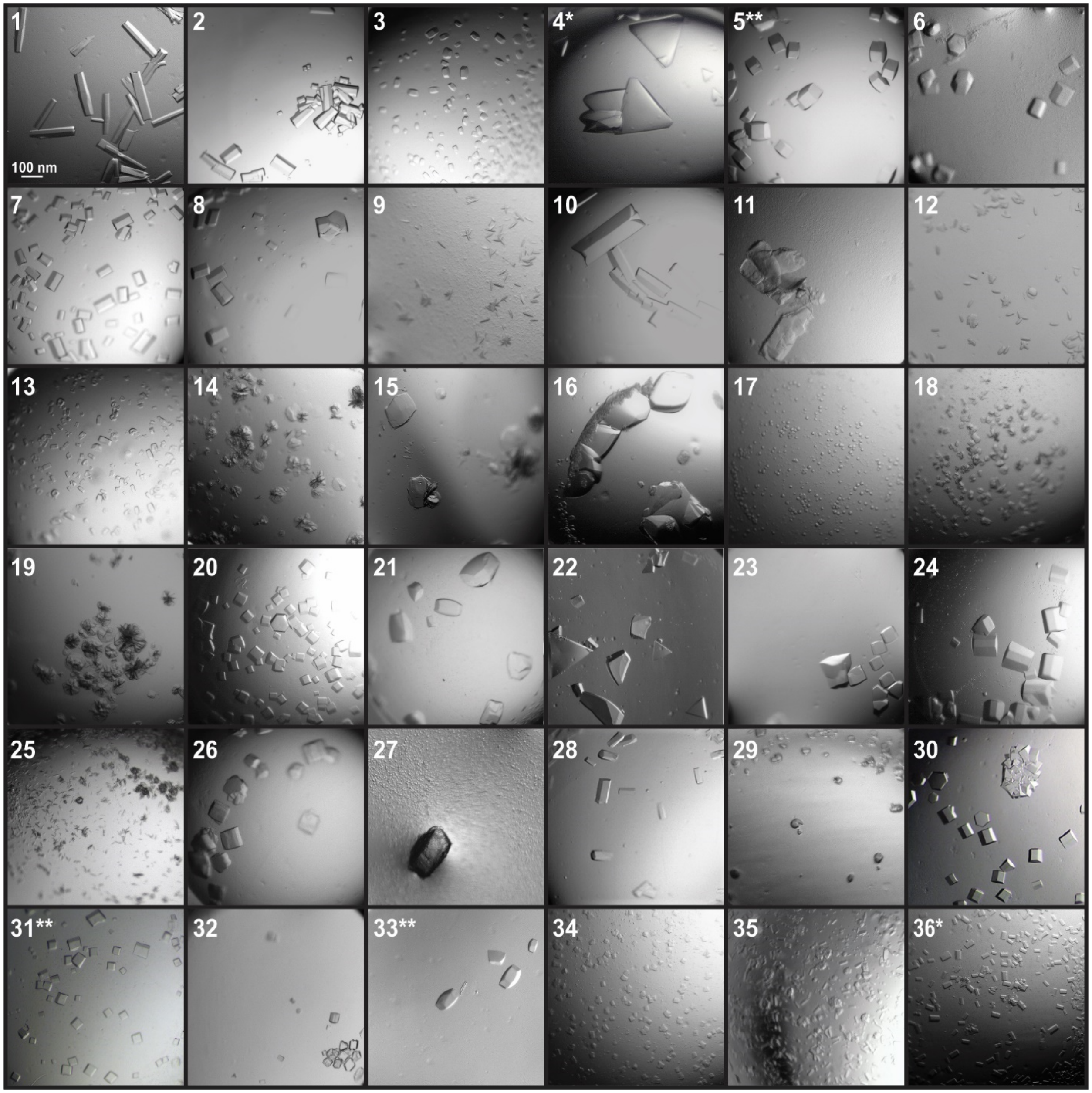

Supplementary Figure 8. Representative bright-field images from the crystallization screen of all 36 immobile Holliday junctions in the 4x6 system. The buffers that correspond to each image are indicated in Supplementary table 7, and the resulting viability of each crystal type used for structure solution, or for those conditions ultimately determined as "fatal", can be found in Supplementary table 8. Junctions with *R*3 symmetry are denoted with an asterisk and junctions with both *R*3and $P3_2$ symmetry are denoted with a double asterisk.

Supplementary Table 8. Summary of the resulting space group and corresponding resolution for all 4x6 crystal structures. All 36 of each junction type are shown, with those determined as “fatal” highlighted in red.

Junction	Space Group	Resolution
1	$P3_2$	3.05
2	$P3_2$	3.15
3	—	—
4	$R3$	3.15
5	$P3_2$	3.15
5	$R3$	3.1
6	—	—
7	$P3_2$	3.1
8	$P3_2$	3
9	—	—
10	$P3_2$	3.1
11	—	—
12	—	—
13	—	—
14	—	—
15	—	—
16	$P3_2$	3.2
17	—	—
18	—	—
19	—	—

Junction	Space Group	Resolution
20	$P3_2$	3.1
21	—	—
22	$P3_2$	3.1
23	$P3_2$	3.1
24	$P3_2$	3.1
25	—	—
26	$P3_2$	3.1
27	—	—
28	$P3_2$	3.1
29	—	—
30	$P3_2$	3.15
31	$P3_2$	4.2
31	$R3$	3.15
32	—	—
33	$P3_2$	3.1
33	$R3$	3.15
34	—	—
35	—	—
36	$R3$	3.05

Supplementary Table 9. The buffer conditions used for the corresponding bright field images shown in Supplementary fig. 9 for the 4x6 system.

Junction	Buffer						
**1**	3	0.05 M Na cacodylate pH 6.5	18 mM $MgCl_2$	0.9 mM spermine	1.8 mM $CoH_{18}N_6$	9% isopropanol	
**2**	14	0.05 M HEPES pH 7.5	20 mM $MgCl_2$	1.0 mM spermine	5% PEG 8000		
**3**	5	0.05 M Na cacodylate pH 7.0	18 mM $MgCl_2$	2.25 mM spermine	0.9 mM $CoH_{18}N_6$	4.5% MPD	
**4**	36	0.05 M Na cacodylate pH 6.5	15 mM $MgCl_2$	5.0 mM spermidine	1.0 mM $CoH_{18}N_6$	2.0 M NaCl	
**5**	35	0.05 M Na cacodylate pH 6.0	200 mM $Ca(CH_3COO)_2$	1.0 mM $CoH_{18}N_6$	2.0 M LiCl		
**6**	20	0.05 M HEPES pH 7.5	15 mM $MgCl_2$	1.0 mM spermidine	10% dioxan		
**7**	35	0.05 M Na cacodylate pH 6.0	200 mM $Ca(CH_3COO)_2$	1.0 mM $CoH_{18}N_6$	2.0 M LiCl		
**8**	34	0.05 M Na cacodylate pH 6.0	200 mM $Ca(CH_3COO)_2$	2.5 M NaCl			
**9**	38	0.05 M TRIS pH 7.5	50 mM $MgCl_2$	1.0 M sodium tartarate			
**10**	26	0.05 M Na cacodylate pH 6.0	200 mM $Ca(CH_3COO)_2$	5% isopropanol			
**11**	33	0.05 M HEPES pH 7.5	15 mM $MgCl_2$	1.0 mM spermine	1.0 M $(NH_4)_2SO_4$		
**12**	33	0.05 M HEPES pH 7.5	15 mM $MgCl_2$	1.0 mM spermine	1.0 M $(NH_4)_2SO_4$		
**13**	32	0.05 M Na cacodylate pH 6.5	10 mM $MgCl_2$	1.5 mM spermine	3.0 M $(NH_4)_2SO_4$		
**14**	29	0.05 M Na cacodylate pH 6.5	10 mM $MgCl_2$	200 mM sodium citrate	5% isopropanol		
**15**	16	0.05 M Na cacodylate pH 6.0	10 mM $MgCl_2$	2.5 mM spermine	5 mM $CaCl_2$	10% isopropanol	
**16**	5	0.05 M Na cacodylate pH 7.0	18 mM $MgCl_2$	2.25 mM spermine	0.9 mM $CoH_{18}N_6$	4.5% MPD	
**17**	31	0.05 M Na cacodylate pH 6.5	20 mM $MgCl_2$	1.0 mM spermine	2.0 M $(NH_4)_2SO_4$		
**18**	14	0.05 M HEPES pH 7.5	20 mM $MgCl_2$	1.0 mM spermine	5% PEG 8000		
**19**	17	0.05 M Na cacodylate pH 7.0	9 mM $MgCl_2$	2.25 mM spermine	1.8 mM $CoH_{18}N_6$	0.9 mM spermidine	5% PEG 400
**20**	25	0.05 M Na cacodylate pH 6.5	30 mM $MgCl_2$	1.0 mM spermine	1.3 M Li_2SO_4		
**21**	23	0.05 M Na cacodylate pH 6.5	2.0 mM spermine	1.0 mM $CoH_{18}N_6$	80 mM $CaCl_2$		
**22**	34	0.05 M Na cacodylate pH 6.0	200 mM $Ca(CH_3COO)_2$	2.5 M NaCl			
**23**	33	0.05 M HEPES pH 7.5	15 mM $MgCl_2$	1.0 mM spermine	1.0 M $(NH_4)_2SO_4$		
**24**	24	0.05 M Na cacodylate pH 6.5	5 mM $MgCl_2$	2.5 mM $CoH_{18}N_6$			
**25**	22	0.05 M Na cacodylate pH 6.5	2.5 mM spermine	18 mM $CaCl_2$	9% 2-propanol		
**26**	23	0.05 M Na cacodylate pH 6.5	2.0 mM spermine	1.0 mM $CoH_{18}N_6$	80 mM $CaCl_2$		
**27**	26	0.05 M Na cacodylate pH 6.0	200 mM $Ca(CH_3COO)_2$	5% isopropanol			
**28**	20	0.05 M HEPES pH 7.5	15 mM $MgCl_2$	1.0 mM spermidine	10% dioxan		
**29**	20	0.05 M HEPES pH 7.5	15 mM $MgCl_2$	1.0 mM spermidine	10% dioxan		
**30**	40	0.05 M Na cacodylate pH 6.0	200 mM $MgCl_2$	2.5 M KCl			
**31**	34	0.05 M Na cacodylate pH 6.0	200 mM $Ca(CH_3COO)_2$	2.5 M NaCl			
**32**	7	0.05 M Na succinate pH 5.5	10 mM $MgCl_2$	2.0 mM $CoH_{18}N_6$	10% isopropanol		
**33**	7	0.05 M Na succinate pH 5.5	10 mM $MgCl_2$	2.0 mM $CoH_{18}N_6$	10% isopropanol		
**34**	34	0.05 M Na cacodylate pH 6.0	200 mM $Ca(CH_3COO)_2$	2.5 M NaCl			
**35**	31	0.05 M Na cacodylate pH 6.5	20 mM $MgCl_2$	1.0 mM spermine	2.0 M $(NH_4)_2SO_4$		
**36**	30	0.05 M Na cacodylate pH 6.5	15 mM $MgCl_2$	10 mM spermine	2.0 M Li_2SO_4		

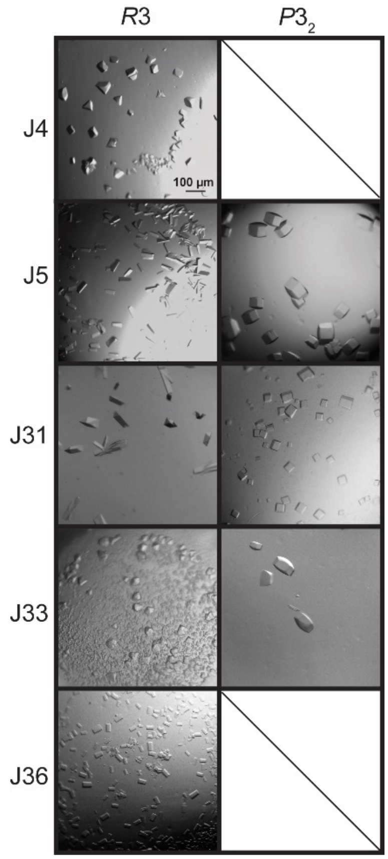

Supplementary Figure 9. Representative bright field images for the five junctions resulting in *R*3 symmetry in the 4x6 crystals. J4, 5, 31, 33, and 36 all yielded *R*3 symmetry (left) which was a significant departure in symmetry from the original 4x6 system (*P*3$_2$). Further, J5, 31, and 33 exhibited both symmetries in a buffer dependent fashion (right). See main text for details.

Supplementary Table 10. The buffer conditions used for the corresponding bright field images shown in Supplementary figure 10 for the 4x6 system comparing the R3 vs P3$_2$ preferences for low and high salt.

Junction	Symmetry	Buffer	Buffer Components				
5	*P*3$_2$	35	0.05 M Na cacodylate pH 6.0	200 mM $Ca(CH_3COO)_2$	1.0 mM $CoH_{18}N_6$	2.0 M LiCl	
31	*P*3$_2$	34	0.05 M Na cacodylate pH 6.0	200 mM $Ca(CH_3COO)_2$	2.5 M NaCl		
33	*P*3$_2$	7	0.05 M Na succinate pH 5.5	10 mM $MgCl_2$	2.0 mM $CoH_{18}N_6$	10% isopropanol	
4	*R*3	9	0.05 M Na cacodylate pH 7.0	20 mM $MgCl_2$	1.0 mM spermine	1.0 mM CoH18N6	15% Ethanol
5	*R*3	18	0.05 M Na cacodylate pH 6.5	10 mM $MgCl_2$	2.5 mM spermine	1 mM $CuSO_4$	10% Isopropanol
31	*R*3	22	0.05 M Na cacodylate pH 6.5	2.5 mM spermine	18 mM $CaCl_2$	9% 2-propanol	
33	*R*3	4	0.05 M Na cacodylate pH 6.5	18 mM $MgCl_2$	2.25 mM spermine	9% Isopropanol	
36	*R*3	48	0.05 M Na cacodylate pH 6.5	10 mM $MgCl_2$	50 mM spermine		

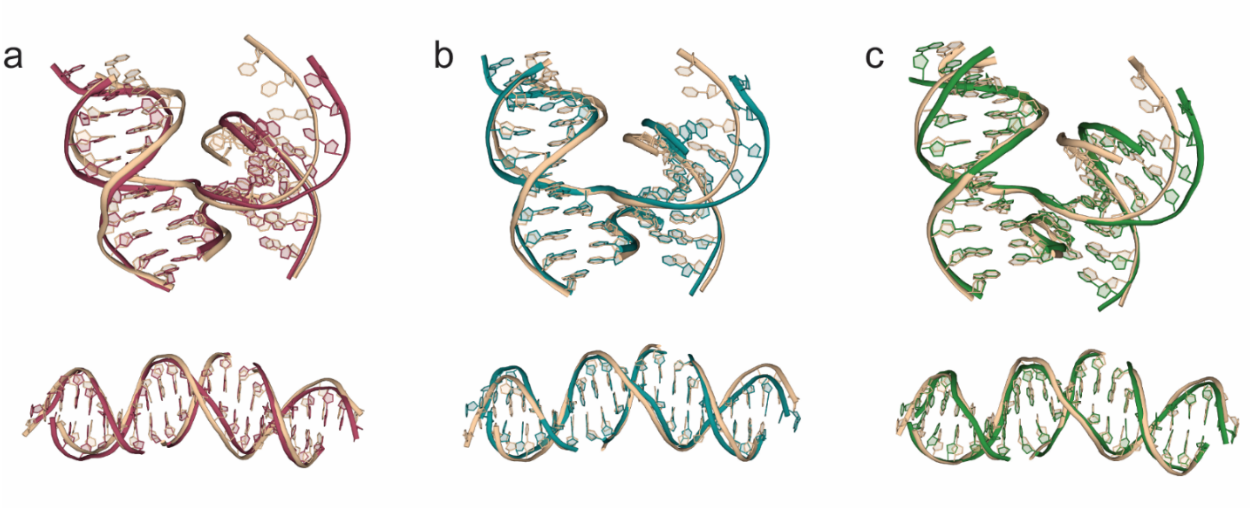

Supplementary Figure 10. Superimposed views of 4x6 junction crystals exhibiting both $P3_2$ and $R3$ symmetry. Both the junction and duplex models are shown for each respective junction. (a-c) J5, J31, and J33, respectively, each contained both $P3_2$ (tan) and $R3$ (green) symmetry in a buffer dependent fashion (see main text for details). Attempts at global alignments of the junction structures reveal the dramatic influence of symmetry as evidenced from the significant misalignment of the right arm of each structure.

Supplementary Table 11. Parallel comparison of the fate of each junction sequence between the 4x5 and 4x6 systems. All 36 junctions are indicated with those that successfully crystallized and solved boxed in green, and those that were ultimately fatal in red to provide context for junctions sharing a common fate in each system. Note that junctions 11, 12, 13, 17, 18, and 27 proved fatal in each motif.

Junction	1	2	3	4	5	6	7	8	9	10	11	12
4x5	green	red	green	red	green	green	green	green	green	green	red	red
4x6	green	green	red	green	green	red	green	green	red	green	red	red

Junction	13	14	15	16	17	18	19	20	21	22	23	24
4x5	red	green	green	green	red	red	green	green	green	green	green	green
4x6	red	red	red	green	red	red	red	green	red	green	green	green

Junction	25	26	27	28	29	30	31	32	33	34	35	36
4x5	green	green	red	green	green	red	green	green	green	green	green	green
4x6	red	green	red	green	red	green	green	red	green	red	red	green

Supplementary Table 12. Unit cell dimensions for each corresponding 4x6 junction crystal. The structures were divided according to their crystal symmetry with the axis lengths indicated. The average and standard deviation for the respective axes of the two symmetries were also calculated and are shown in bold in the final row. Angles corresponding to both *P*32 and *R*3 crystals were $\alpha = \beta = 90°$ $\gamma = 120°$, irrespective of symmetry.

**4x6 *P*32**		
Junction	a,b	c
1	68.44	55.68
2	69.11	56.4
5	68.17	55.46
7	68.01	54.15
8	68.3	54.28
10	67.74	53.48
16	68.15	53.79
20	68.07	56.06
22	68.55	55.36
23	68.63	55.96
24	68.1	57.3
26	68.17	55.08
28	67.9	59.51
30	68.1	52.77
31	68.76	55.21
33	68.39	60.35
**Average**	**68.29±0.34**	**55.68±1.96**

**4x6 *R*3**		
Junction	a,b	c
4	115.15	48.66
5	114.78	49.62
31	116.08	49.43
33	113.87	50.81
36	114.61	50.35
**Average**	**114.90±0.72**	**49.77±0.75**

.

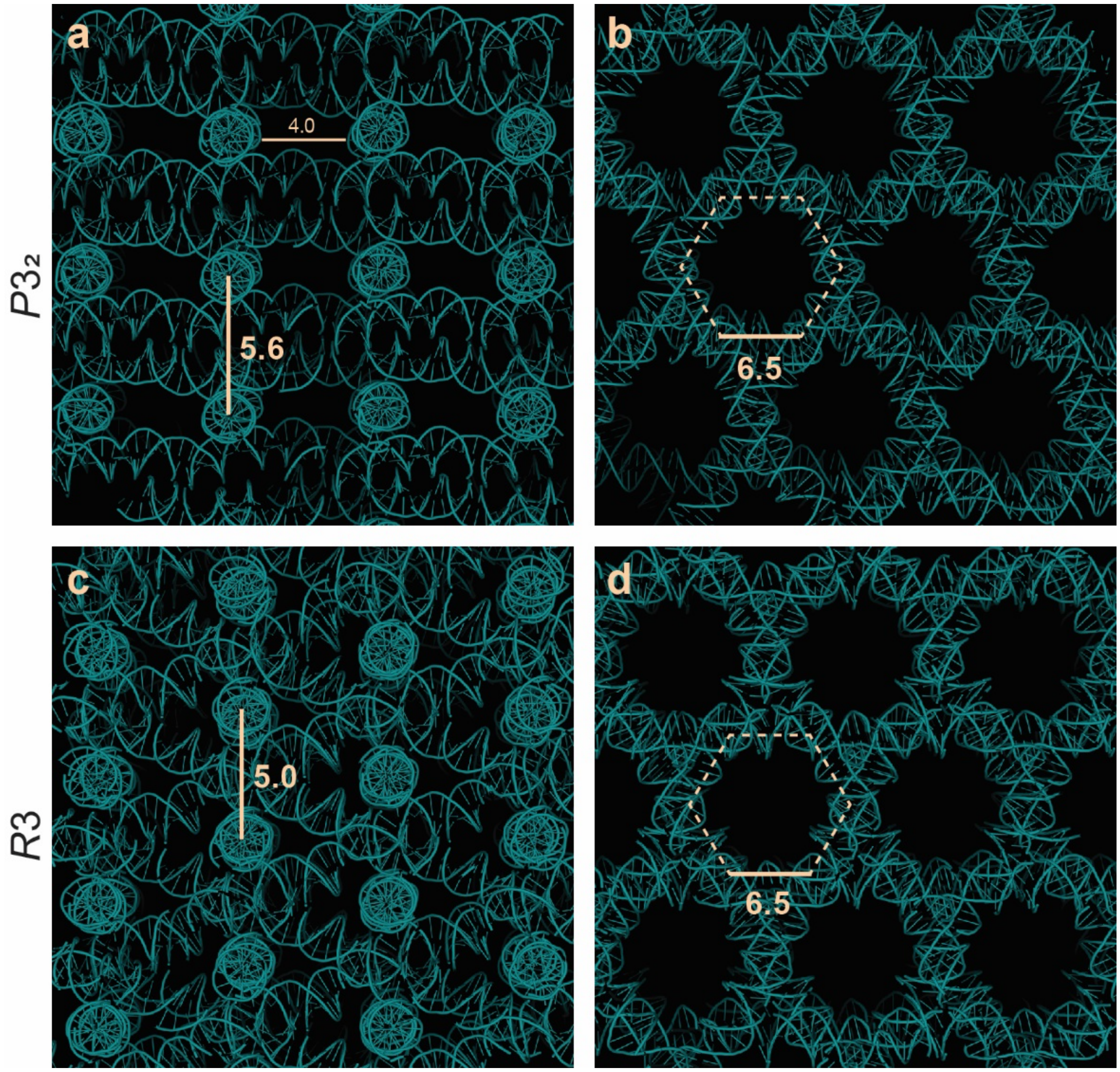

Supplementary Figure 11. Symmetry related duplexes show the full crystal lattice for both symmetries observed in the 4x6 motif. Panels a) and b) correspond to the $P3_2$ symmetric lattices and are rotated 90° with respect to one another. (a) The resulting periodic cavities in the 4x6 system with $P3_2$ symmetry are evident in (a) with a height of 5.6 nm along the edge of the cavity shown in this view, resulting from the distance from the top to the bottom of each 4 layer block. This height is also in good agreement to the average c-axis of the $P3_2$ lattices (55.68±1.96). (b) View oriented 90° with respect to (a) revealing a view of the cavity containing six sides of 6.5 nm along each edge. The corresponding cavity volumes were calculated as a hexagonal prism with an edge length of 6.5 nm and a height of 5.6 nm. See main text for additional details. Panels c) and d) correspond to the $P3_2$ lattices and are rotated 90° with respect to one another. Panels (c) and (d) correspond to the *R*3 symmetric lattices and are rotated 90° with respect to one another. The resulting cavities with *R*3 symmetry contain a height of 5.0 nm which is in good agreement to the average c-axis of the *R*3 lattices (49.77±0.75). (d) View oriented 90° with respect to (c) showing the cavity also containing six sides of 6.5 nm along each edge. The corresponding cavity volumes were also calculated as hexagonal prisms with an edge length of 6.5 nm and a height of 5.0 nm. See main text for additional details.

Supplementary Table 13. Calculated interduplex angles (IDA) corresponding to each junction structure for the 4x6 system. The listed junctions are divided according to crystal symmetry with each respective angle listed. The average and standard deviation for the respective angles of the two symmetries were also calculated and are shown in bold in the final row.

**4x6**			
***P*32**		***R*3**	
Junction	Angle	Junction	Angle
1	54.34	4	61.72
2	55.71	5	57.34
5	52.48	31	59.36
7	56.25	33	55.49
8	53.25	36	57.92
10	55.15	**58.37±2.33**	
16	52.44		
20	52.56		
22	57.7		
23	54.61		
24	54.73		
26	55.35		
28	54.14		
30	54.66		
31	54.53		
33	55.67		
**54.60±1.44**			

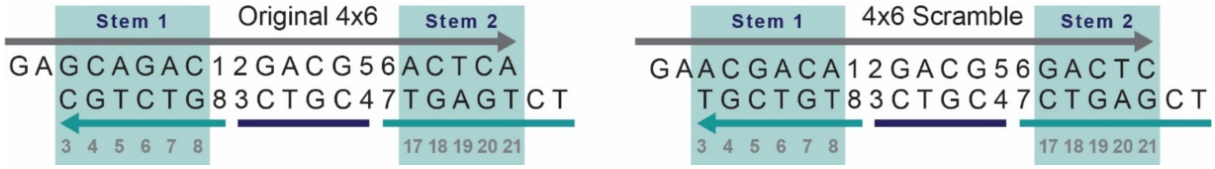

Supplementary Figure 12. Duplex comparison of the original and scrambled 4x6 sequences. The flanking region between the Holliday junctions and sticky ends were changed to contain the opposite nitrogeneous base and base pair. The corresponding scrambled bases are denoted with a number corresponding to a position for the 21 bp duplex, starting at the 5' of the linear S2 strand.

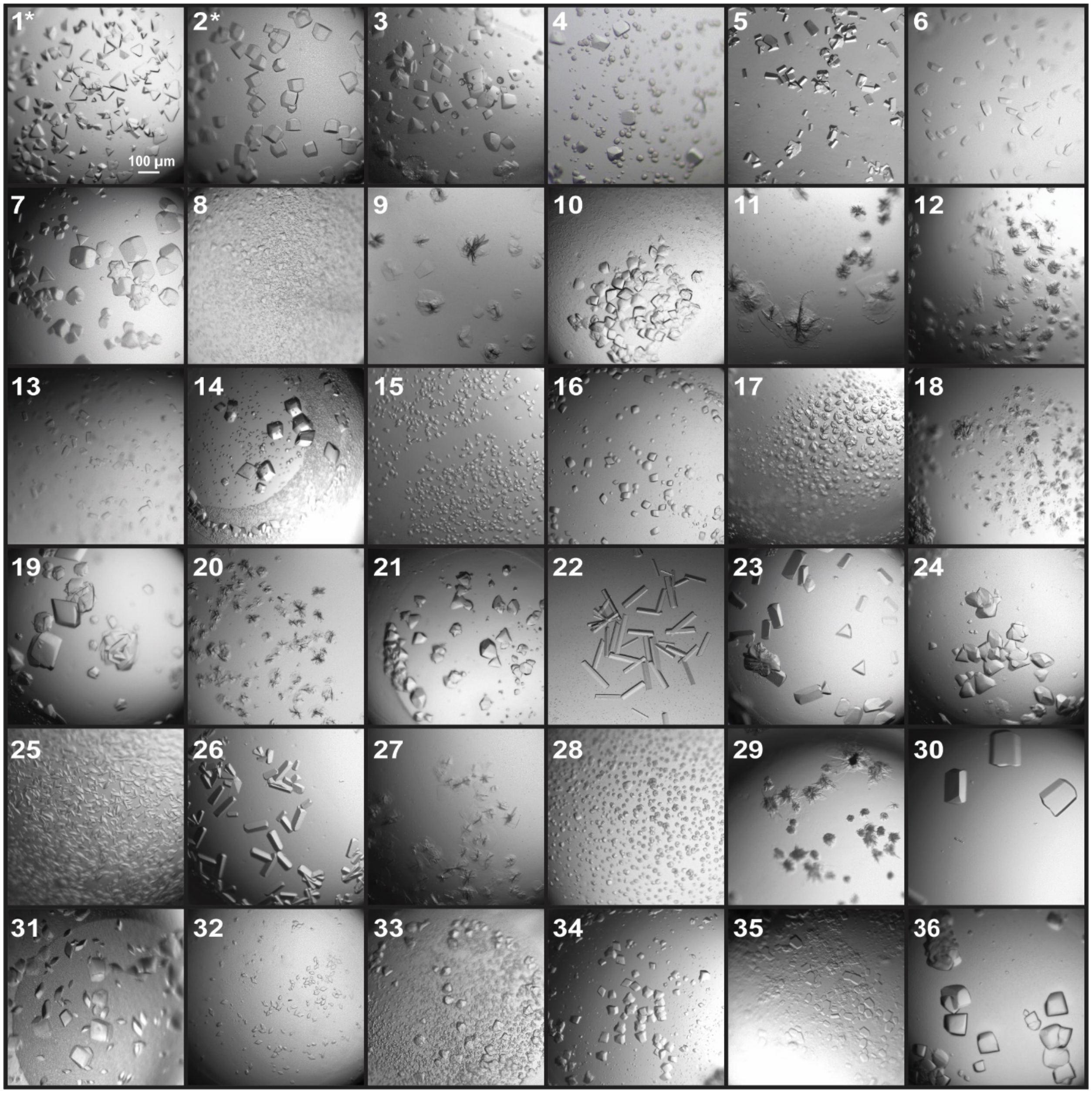

Supplementary Figure 13. Representative bright-field images from the crystallization screen of all 36 immobile Holliday junctions in the 4x6 "scramble" system. The buffers that correspond to each image are indicated in Supplementary table 13, and the resulting viability of each crystal type used for structure solution, or for those conditions ultimately determined as "fatal", can be found in Supplementary table 11. The junctions denoted with an asterisk crystallized with $P3_2$ symmetry.

Supplementary Table 14. Buffers used for representative bright field image shown in supplementary figure 14 for the 4x6 scramble system.

**Junction**	Buffer						
**1**	3	0.05 M Na Cacodylate pH 6.5	18 mM $MgCl_2$	0.9 mM spermine	1.8 mM $CoH_{18}N_6$	9% isopropanol	
**2**	1	0.05 M HEPES pH 7.5	80 mM $MgCl_2$	2.5 mM spermine			
**3**	2	0.05 M Na Cacodylate pH 6.0	18 mM $MgCl_2$	2.25 mM spermine	1 mM $CuSO_4$	9% isopropanol	
**4**	10	0.05 M Na Cacodylate pH 7.0	5 mM $MgCl_2$	1.0 mM spermine	10% tert-butanol		
**5**	19	0.05 M Na Cacodylate pH 6.0	20 mM $MgCl_2$	1.0 mM spermine	2 mM $CaCl_2$	10% MPD	
**6**	44	0.05 M Na succinate pH 5.5	20 mM $MgCl_2$	0.5 mM spermine	3.0 M $(NH_4)_2SO_4$		
**7**	10	0.05 M Na Cacodylate pH 7.0	5 mM $MgCl_2$	1.0 mM spermine	10% tert-butanol		
**8**	19	0.05 M Na Cacodylate pH 6.0	20 mM $MgCl_2$	1.0 mM spermine	2 mM $CaCl_2$	10% MPD	
**9**	17	0.05 M Na Cacodylate pH 7.0	9 mM $MgCl_2$	2.25 mM spermine	1.8 mM $CoH_{18}N_6$	0.9 mM spermidine	5% PEG 400
**10**	9	0.05 M Na acodylate pH 7.0	20 mM $MgCl_2$	1.0 mM spermine	1.0 mM $CoH_{18}N_6$	15% ethanol	
**11**	15	0.05 M Na Cacodylate pH 6.0	20 mM $MgCl_2$	2.5 mM spermine	5% PEG 4000		
**12**	32	0.05 M Na Cacodylate pH 6.5	10 mM $MgCl_2$	1.5 mM spermine	3.0 M $(NH_4)_2SO_4$		
**13**	32	0.05 M Na Cacodylate pH 6.5	10 mM $MgCl_2$	1.5 mM spermine	3.0 M $(NH_4)_2SO_4$		
**14**	9	0.05 M Na Cacodylate pH 7.0	20 mM $MgCl_2$	1.0 mM spermine	1.0 mM $CoH_{18}N_6$	15% ethanol	
**15**	42	0.05 M Na Cacodylate pH 6.0	15 mM $MgCl_2$	5.0 mM spermidine	2.0 M Li_2SO_4		
**16**	10	0.05 M Na Cacodylate pH 7.0	5 mM $MgCl_2$	1.0 mM spermine	10% tert-butanol		
**17**	31	0.05 M Na Cacodylate pH 6.5	20 mM $MgCl_2$	1.0 mM spermine	2.0 M $(NH_4)_2SO_4$		
**18**	15	0.05 M Na Cacodylate pH 6.0	20 mM $MgCl_2$	2.5 mM spermine	5% PEG 4000		
**19**	1	0.05 M HEPES pH 7.5	80 mM $MgCl_2$	2.5 mM spermine			
**20**	13	0.05 M TRIS pH 8.0	10 mM $MgCl_2$	1.0 mM $CoH_{18}N_6$	20% ethanol		
**21**	4	0.05 M Na Cacodylate pH 6.5	18 mM $MgCl_2$	2.25 mM spermine	9% Isopropanol		
**22**	11	0.05 M Na Cacodylate pH 7.0	30 mM $MgCl_2$	2.5 mM spermine	5% PEG 400		
**23**	1	0.05 M HEPES pH 7.5	80 mM $MgCl_2$	2.5 mM spermine			
**24**	4	0.05 M Na Cacodylate pH 6.5	18 mM $MgCl_2$	2.25 mM spermine	9% Isopropanol		
**25**	3	0.05 M Na Cacodylate pH 6.5	18 mM $MgCl_2$	0.9 mM spermine	1.8 mM $CoH_{18}N_6$	9% isopropanol	
**26**	3	0.05 M Na Cacodylate pH 6.5	18 mM $MgCl_2$	0.9 mM spermine	1.8 mM $CoH_{18}N_6$	9% isopropanol	
**27**	37	0.05 M Na Cacodylate pH 6.5	200 mM $MgCl_2$	100 mM NaCl	20% PEG 1000		
**28**	15	0.05 M Na Cacodylate pH 6.0	20 mM $MgCl_2$	2.5 mM spermine	5% PEG 4000		
**29**	14	0.05 M HEPES pH 7.5	20 mM $MgCl_2$	1.0 mM spermine	5% PEG 8000		
**30**	12	0.05 M Na Cacodylate pH 6.5	100 mM $MgCl_2$	2.0 mM $CoH_{18}N_6$	5% isopropanol		
**31**	2	0.05 M Na Cacodylate pH 6.0	18 mM $MgCl_2$	2.25 mM spermine	1 mM $CuSO_4$	9% isopropanol	
**32**	44	0.05 M Na succinate pH 5.5	20 mM $MgCl_2$	0.5 mM spermine	3.0 M $(NH_4)_2SO_4$		
**33**	34	0.05 M Na Cacodylate pH 6.0	200 mM $Ca(CH_3COO)_2$	2.5 M NaCl			
**34**	8	0.05 M Na Cacodylate pH 6.0	20 mM $MgCl_2$	1.0 mM spermine	15% ethanol		
**35**	31	0.05 M Cacodylate pH 6.5	20 mM $MgCl_2$	1.0 mM spermine	2.0 M $(NH_4)_2SO_4$		
**36**	5	0.05 M Na Cacodylate pH 7.0	18 mM $MgCl_2$	2.25 mM spermine	0.9 mM $CoH_{18}N_6$	4.5% MPD	

Supplementary Table 15. Summary of the resulting space group and corresponding resolution for all 4x6 "scramble" crystal structures. All 36 of each junction type are shown, with those determined as "fatal" highlighted in red.

Junction	Space Group	Resolution	Junction	Space Group	Resolution
1	$P3_2$	3.05	19	*R*3	3.00
2	$P3_2$	3.05	20	—	—
3	*R*3	2.85	21	*R*3	3.05
4	—	—	22	*R*3	3.15
5	*R*3	3.10	23	*R*3	3.00
6	—	—	24	*R*3	2.90
7	*R*3	3.00	25	—	—
8	*R*3	3.05	26	*R*3	3.00
9	—	—	27	—	—
10	*R*3	2.80	28	—	—
11	—	—	29	—	—
12	—	—	30	*R*3	3.10
13	—	—	31	*R*3	2.95
14	*R*3	3.00	32	—	—
15	—	—	33	*R*3	3.10
16	*R*3	3.00	34	*R*3	3.00
17	—	—	35	—	—
18	—	—	36	*R*3	2.70

Supplementary Table 16. Parallel comparison of the fate of each junction sequence between the 4x5, 4x6, and 4x6 scrambled sequence systems. All 36 junctions are indicated with those that successfully crystallized and solved boxed in green, and those that were ultimately fatal in red to provide context for junctions sharing a common fate in each system. Note that junctions 11, 12, 13, 17, 18, and 27 proved fatal in each motif, further substantiating the potential role that each of these respective junctions on the ability of each motif to crystallize.

**Junction**	**1**	**2**	**3**	**4**	**5**	**6**	**7**	**8**	**9**	**10**	**11**	**12**
4x5												
4x6												
4x6 Scramble												

**Junction**	**13**	**14**	**15**	**16**	**17**	**18**	**19**	**20**	**21**	**22**	**23**	**24**
4x5												
4x6												
4x6 Scramble												

**Junction**	**25**	**26**	**27**	**28**	**29**	**30**	**31**	**32**	**33**	**34**	**35**	**36**
4x5												
4x6												
4x6 Scramble												

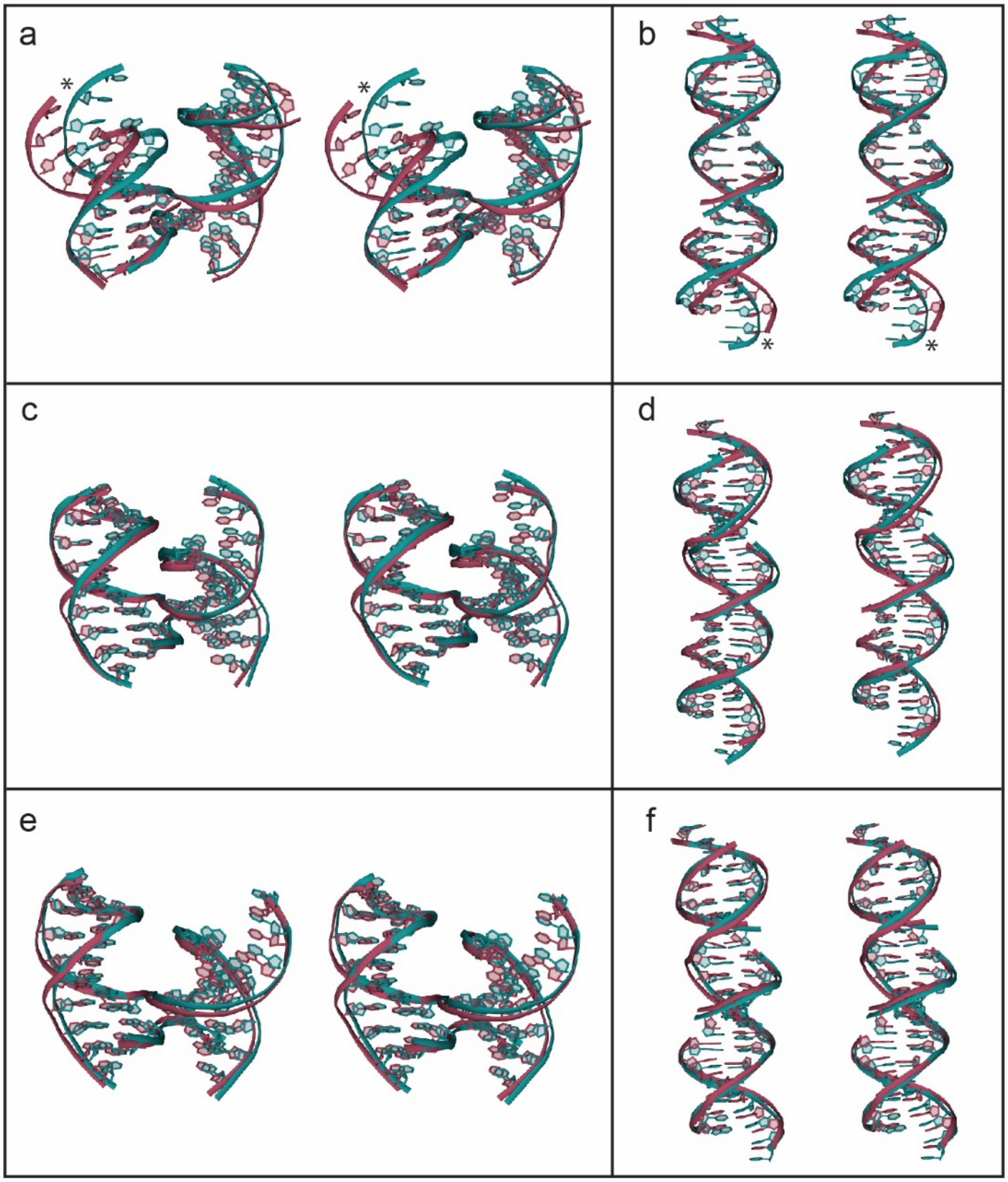

Supplementary Figure 14. Stereoviews of superimposed structures representing unique or consistent symmetries between the 4x6 and 4x6 scrambled structures. (a) and (b) contain stereoviews of the junction and duplex structures, respectively. The superimposed structures compare the original (teal) versus scramble (red) 4x6 variations of J16 containing $P3_2$ symmetry in the original sequence and $R3$ in the scramble. The sites where the structures have a significant departure from one another are indicated with an asterisk in the both the junction and duplex structures. (c) and (d) compare the original (teal) versus scramble (red) 4x6 variations of J2 which both contained $P3_2$ symmetry. (e) and (f) compare the original (teal) versus scramble (red) 4x6 variations of J31 which both contained $R3$ symmetry. No significant structural differences between (c) and (d) with $P3_2$ symmetry, and (e) and (f) with $R3$ symmetry were observable, regardless of the stem sequence.

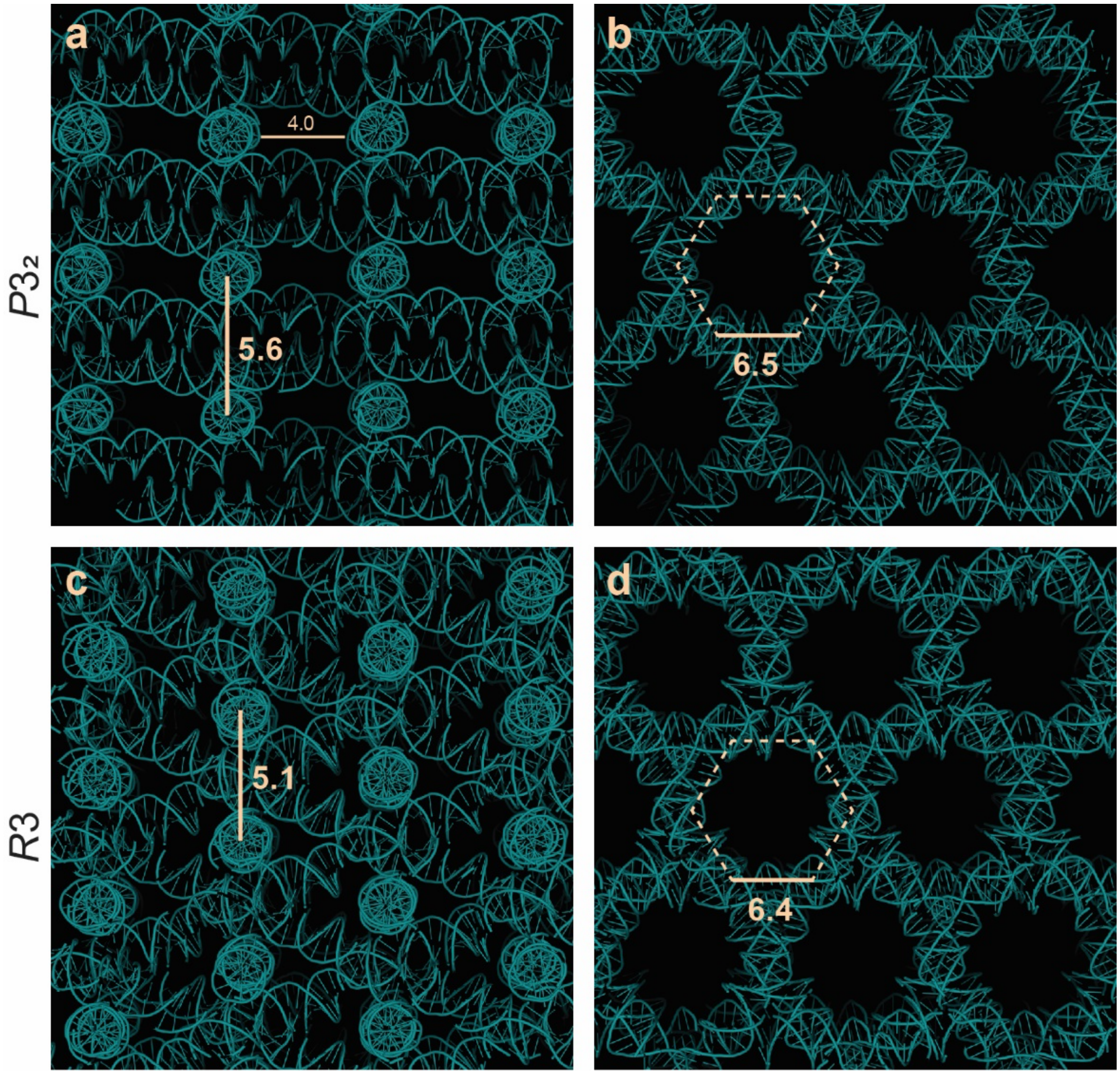

Supplementary Figure 15. Symmetry related duplexes show the full crystal lattice for both symmetries observed in the 4x6 "scramble" motif. Panels a) and b) correspond to the $P3_2$ symmetric lattices and are rotated 90° with respect to one another. (a) The resulting periodic cavities in the 4x6 scramble system containing $P3_2$ symmetry are in good agreement with the original sequence motif with a height of 5.6 nm, resulting from the distance from the top to the bottom of each 4 layer block. This height also appropriately matches the average c-axis of the $P3_2$ lattices (55.81±0.02). (b) View oriented 90° with respect to (a) again revealing a view of the cavity containing six sides of 6.5 nm along each edge. The corresponding cavity volumes were calculated as a hexagonal prism with an edge length of 6.5 nm and a height of 5.6 nm. See main text for additional details. Panels (c) and (d) correspond to the *R*3 symmetric lattices and are rotated 90° with respect to one another. The resulting cavities with *R*3 symmetry contain a height of 5.1 nm which corresponding to the average c-axis of the *R*3 lattices (51.10±0.75). (d) View oriented 90° with respect to (c) showing the cavity also containing six sides with a slightly retracted length of 6.4 nm along each edge. The corresponding cavity volumes were also calculated as hexagonal prisms with an edge length of 6.4 nm and a height of 5.1 nm. See main text for additional details.

Supplementary Table 17. Unit cell dimensions for each corresponding 4x6 "scramble" junction crystal. The structures were divided according to their respective crystal symmetry with the axis lengths indicated. The average and standard deviation for the axes of the two symmetries were also calculated and are shown in bold in the final row. Angles corresponding to both *P*32 and *R*3 crystals were α = β = 90° ɣ = 120°, irrespective of symmetry.

**4x6 Scramble *P*32**		
Junction	a,b	c
1	67.97	55.83
2	68.27	55.79
**Average**	**68.12±0.15**	**55.81±0.02**

**4x6 Scramble *R*3**		
Junction	a,b	c
3	112.21	50.99
5	113.26	49.9
7	113.72	52.05
8	113.17	50.78
10	113.08	52.13
14	112.75	51.06
16	113.31	52.18
19	111.97	50.99
21	113.38	51.31
22	113.02	49.34
23	114.26	51.47
24	112.59	51.86
26	112.09	51.13
30	114.7	50.46
31	113.11	50.39
33	113.57	51.8
34	111.8	51.3
36	112.71	50.62
**Average**	**113.04±0.74**	**51.10±0.75**

.

Supplementary Table 18. Calculated interduplex angles (IDA) corresponding to each junction structure for the 4x6 "scrambled" sequence system. The listed junctions are divided according to crystal symmetry with each respective angle listed. The average and standard deviation for the respective angles of the two symmetries were also calculated and are shown in bold in the final row.

**Scrambled 4x6**			
***P*32**		***R*3**	
Junction	Angle	Junction	Angle
1	57.06	3	60.14
2	59.03	5	58.79
**58.05±1.39**		7	59.93
		8	61.28
		10	62.89
		14	62.1
		16	61.34
		19	60.24
		21	63.45
		22	60.2
		23	60.83
		24	60.47
		26	62.44
		30	60.95
		31	60.46
		33	61.6
		34	59.41
		36	61.54
		**61.00±1.21**	

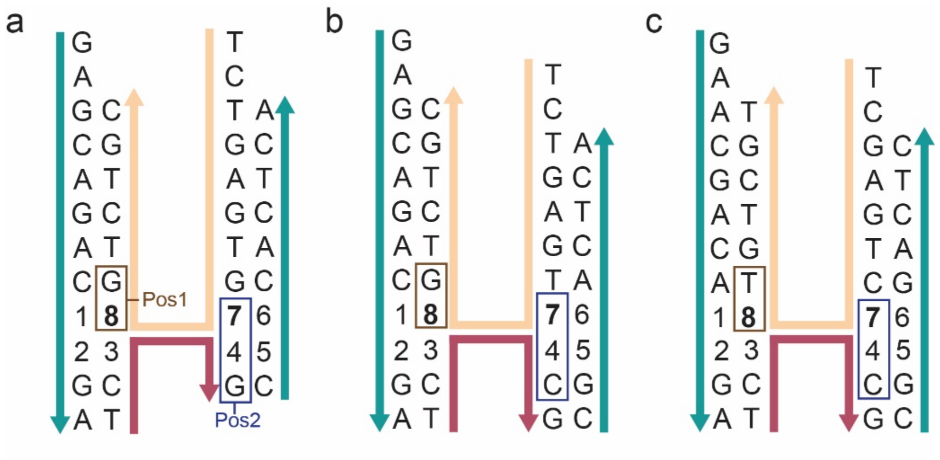

Supplementary Figure 16. Definition of ion positions 1 and 2. The nearest bases to the ions, responsible for coordinating them are boxed in brown and blue, corresponding to the conserved positions 1 and 2 within the (a) 4x5, (b) 4x6, (c) 4x6 scramble systems.

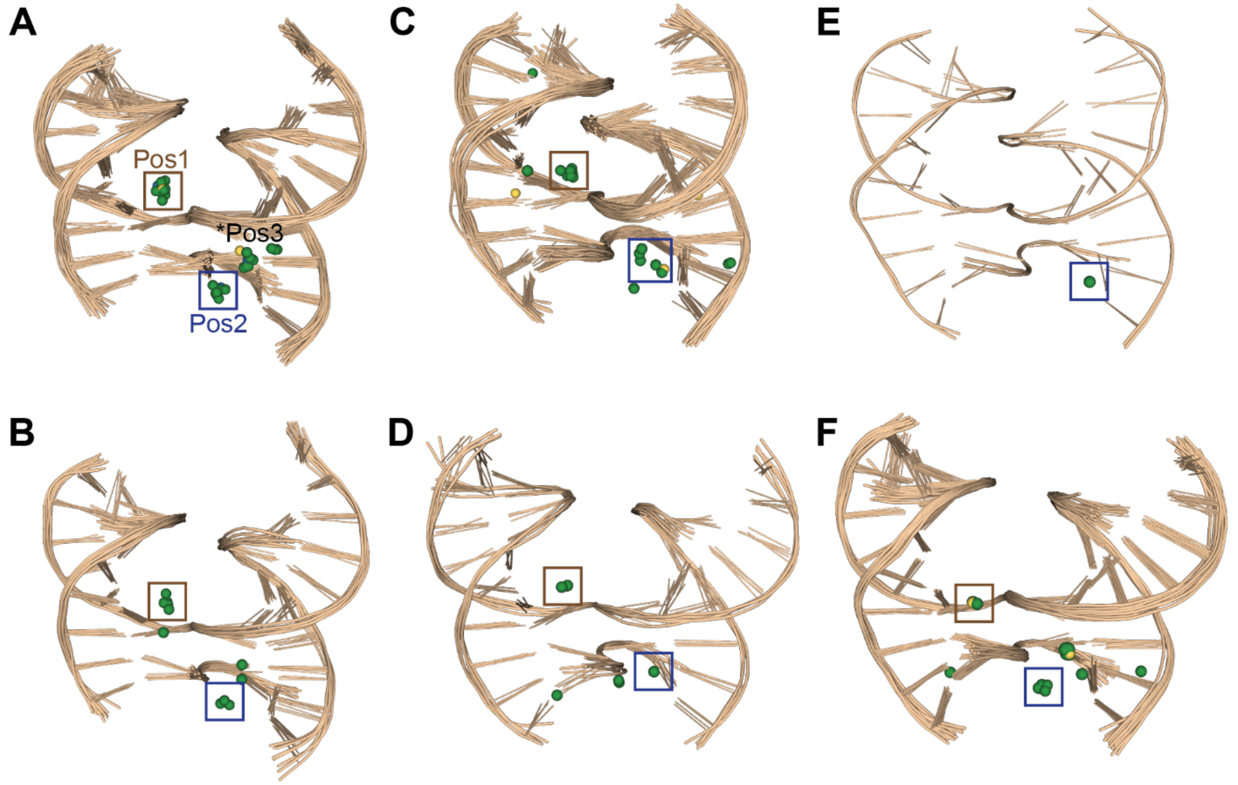

Supplementary Figure 17. Conserved binding sites are consistent regardless of crystal symmetry. The superimposed models in each panel are comprehensive alignments of all structures within a given system and unique space group. The consensus locations of the ions (Pos1 and Pos2) are evident and are boxed in brown and blue, respectively. (a) 4x5 $P3_221$ with Pos1 and Pos2 shown in the boxed regions. The 4x5 crystals containing $P3_221$ symmetry did contain a regular clustering of ions (*Pos3) not immediately proximal to the junction, and did not appear to require junction related bases for binding. This site did not appear regularly in the majority of the other structures. (b) 4x5 $P3_2$, (c) 4x6 $P3_2$, (d) 4x6 $R3$, (e) 4x6 scramble $P3_2$, and (f) $R3$. Arsenic (green), magnesium (yellow), and cobalt (blue), are represented as spheres.

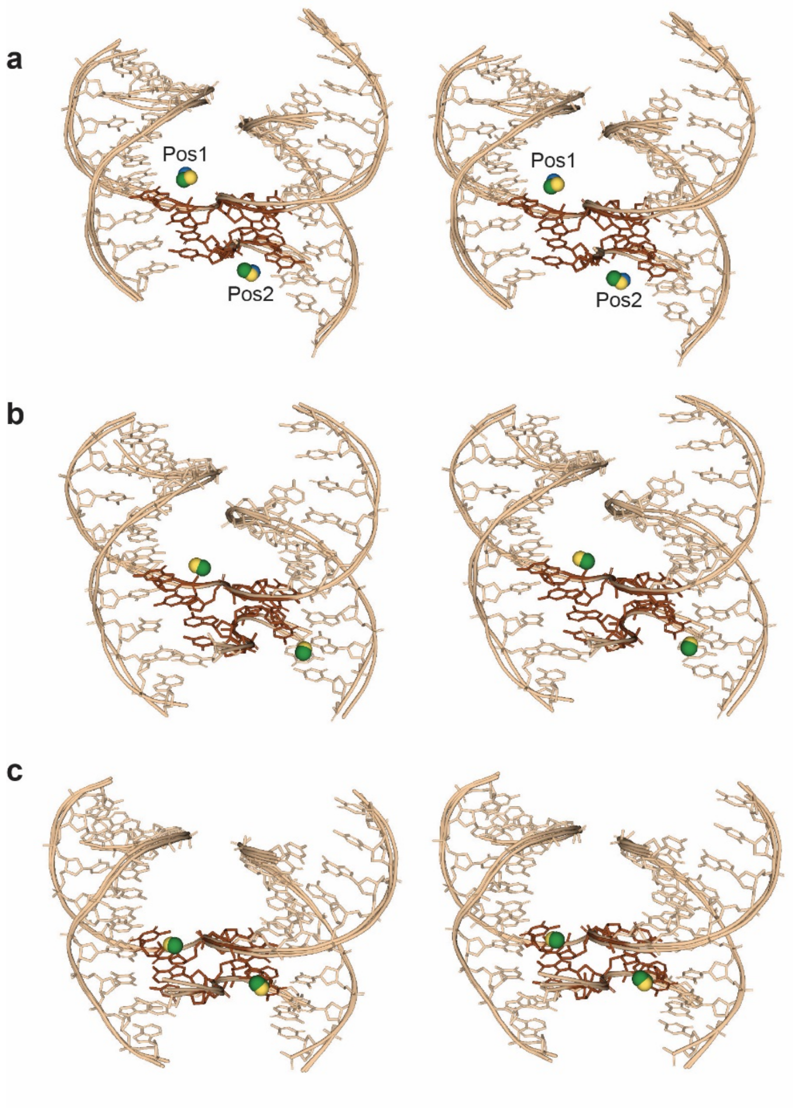

Supplementary Figure 18. Stereoviews of superimposed structures representing conserved ion binding sites in each system. (a) 4x5 junctions 34, 25, and 29, (b) 4x6 junctions 1 and 22, and (c) 4x6 scramble junctions 3, 16, and 23 each represent the conserved ion binding sites (Pos1 and Pos2) in each system. Positions 1 and 2 are located at opposing corners of each junction crossover where the bases that participate in coordination within the junction, and those immediately adjacent to it, are highlighted in dark brown. Arsenic (green), magnesium (yellow), and cobalt (blue), are represented as spheres.

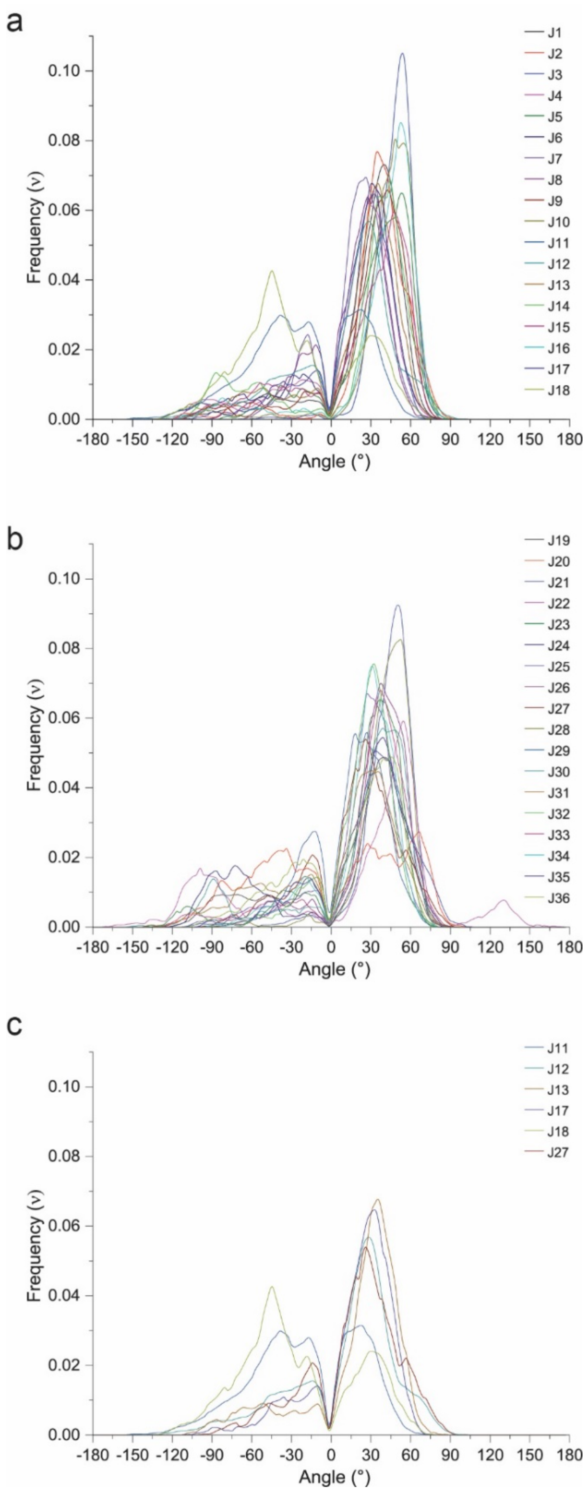

Supplementary Figure 19. Histograms of J_{twist} interhelical angle populations in MD simulations of all 36 immobile HJs (a, b) and (c) of non-crystallizing junctions.

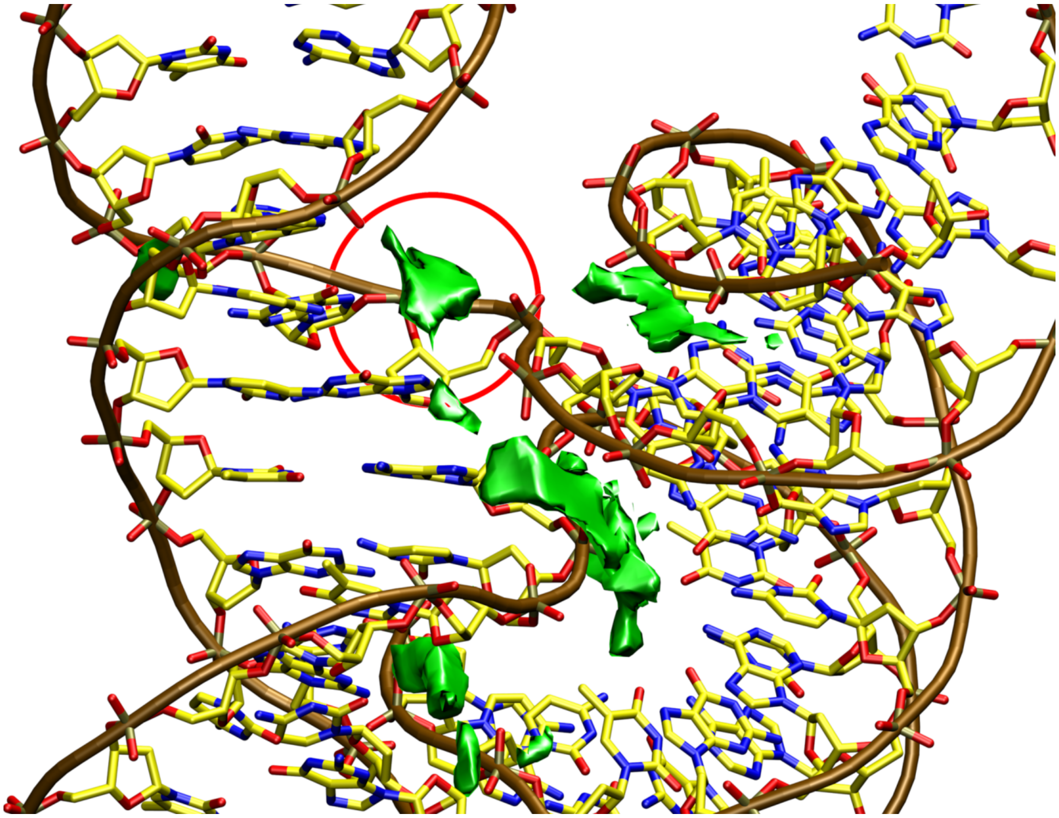

Supplementary Figure 20 . Classical molecular interaction potential (CMIP) calculation[1] of the J1 crystal structure. Areas with large electronegative potential are indicated with green density surfaces. The site where we observe binding of cations in MD simulations and of both cations and anions in experiments is highlighted with a red circle. The CMIP relies on the electrostatic potential (ESP) calculation of the solute in implicit Poisson–Boltzman (PB) solvent.

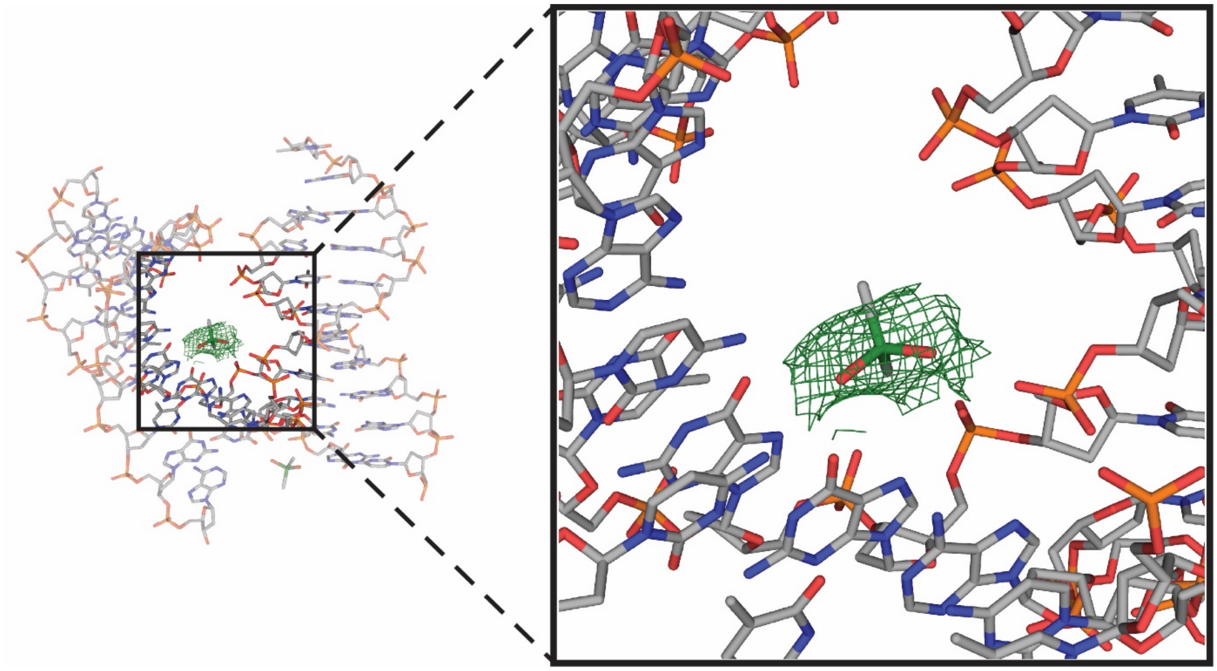

Supplementary Figure 21. Electron density surrounding the cacodylate ion. J1 in the 4x6 system, in $2F_o$ - F_c electron density (contoured at $\boldsymbol{\sigma}$=2.1) displayed which corresponds to the cacodylate ion, that helps facilitate junction crystallization. Atoms are indicated using the following: carbon (gray), nitrogen (blue), oxygen (red), phosphate (orange), and arsenic (green). Arsenic atoms are only added to all crystal structures due to inadequate density coverage in the majority of the structures in the work.

Supplementary Table 19. Median values of the J_{twist} interhelical angles in MD simulations of all 36 immobile HJs. The last column shows the fraction of simulation frames in which the interhelical angle was in the region of 40 to 80 degrees, which corresponds to typical angles found in crystallographic structures. All fatal junction fractions are indicated in red.

Junction	Median	Absolute Deviation	Fraction of Frames (40°-80°)
J1	36.6007	11.9582	0.419005
J2	39.4901	11.0373	0.479068
J3	50.8481	8.3512	0.765644
J4	26.7199	14.6439	0.235714
J5	42.0833	13.972	0.535072
J6	28.9036	14.1006	0.254897
J7	23.9879	12.0694	0.167544
J8	23.6242	14.3494	0.163222
J9	38.2371	13.2068	0.462451
J10	48.5093	10.1487	0.695056
J11	-17.2115	32.1509	0.0530195
J12	23.4316	17.9589	0.202698
J13	31.5024	13.8219	0.311202
J14	38.5286	13.7155	0.464235
J15	37.004	17.6937	0.4567
J16	48.5341	10.7761	0.670156
J17	27.177	13.9075	0.234848
J18	-32.3028	28.7758	0.0959115
J19	29.4304	20.3429	0.336924
J20	7.1505	45.7622	0.280223
J21	47.7136	9.2792	0.689321
J22	43.7266	19.7762	0.469403
J23	34.8739	13.3523	0.390064
J24	32.8885	20.938	0.371806
J25	29.0887	12.885	0.265216
J26	39.379	11.9413	0.484283
J27	25.4667	17.9383	0.259922
J28	46.2497	9.90815	0.650907
J29	21.3262	16.416	0.180386
J30	36.7675	15.2875	0.439613
J31	22.5546	24.3442	0.239828
J32	29.7622	11.7287	0.253412
J33	37.8457	13.3639	0.445836
J34	31.3663	12.1428	0.300252
J35	35.6674	17.9577	0.400221
J36	27.7489	21.9135	0.324571

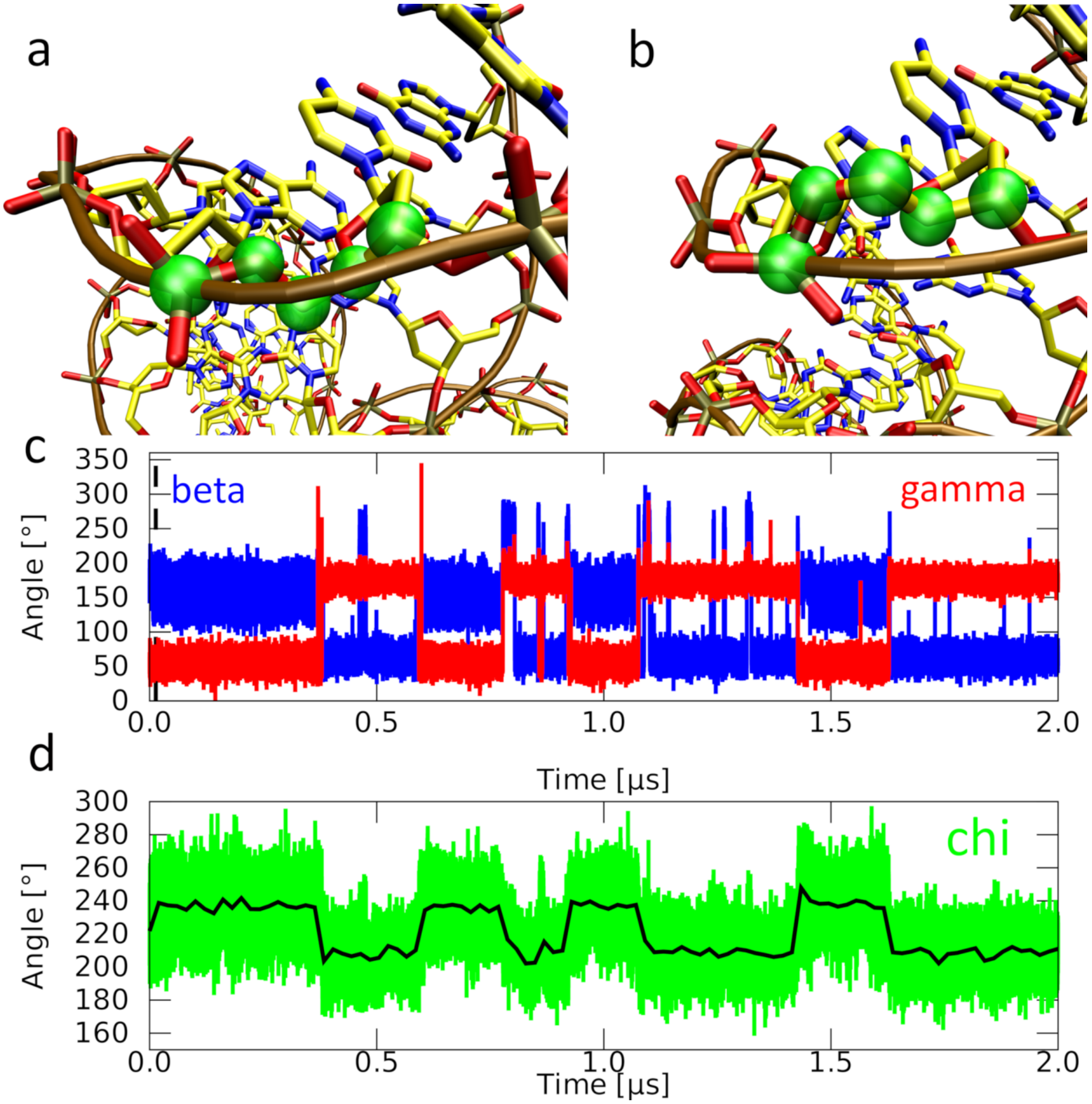

Supplementary Figure 22. **Unusual β/γ conformational states in selected MD simulation of J5 using the parmbsc1 DNA force field.** One specific backbone suite is shown (a T→C step from J5). The same behavior was observed in all HJ simulations when parmbsc1 force field was used. (a, b) One of the nucleotides in the J5 structure which undergoes periodic flips between the native trans/g+ (a) and probably spurious g+/trans (b) conformational states of the β/γ backbone dihedrals. The affected backbone atoms are highlighted with green spheres. (c) Time development of the flips in one of the MD simulations. Parmbsc1 produces a rather large population of the β/γ=g+/trans backbone conformational state which is not supported by our experiments or, to our knowledge, by B-DNA experimental structures. d) The backbone flips are also correlated with transitions of the χ angle of the affected base between *high-anti* region, which is native for B-DNA, and *anti* region characteristic for A-form duplexes. The black line indicates the data trend.

Supplementary Discussion 1. HJ simulations using the parmbsc1 DNA force field.
Here, we have re-simulated a selected set of both universally crystallizing (J1, J5) and non-crystallizing (J11, J13) HJs with the parmbsc1 DNA force field. With the exception of the utilized DNA force-field, the conditions were identical to the OL15 simulations described in the main text. We ran two 2-μs-long simulations for each of the four HJs, obtaining a cumulative length of new simulations of 16 μs. However, we observed long-lived β/γ=g+/trans states in all of the parmbsc1 simulations. The g+/trans conformation of the β/γ dihedrals is not supported by the experimental structures of the HJ, nor is it to our knowledge generally associated with any B-form DNA structures. The overall average population of these states in our dataset was ~5% for internal nucleotides, however, their distribution was very uneven across the DNA strands. For some internal nucleotides, they reached populations as high as ~40%. These states occurred within the helical arms of the HJ as well as its central region. Supplementary Fig. 22 shows a typical example of such behavior. Although the result might be initially surprising, the same problem has been reported recently by another group for B-DNA parmbsc1 simulations.[2] Consistent with this study, we also observe that the β/γ=g+/trans states affect the details of the B-DNA conformation. Based upon our result, we decided to not re-simulate the whole HJ set with parmbsc1 and base the results instead on the OL15 simulations as the β/γ=g+/trans states are absent with OL15.

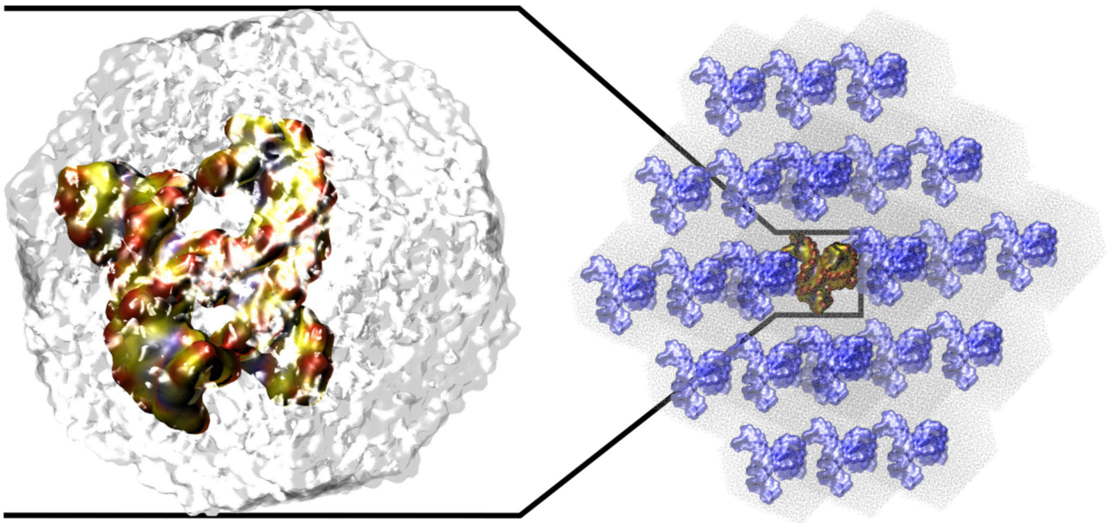

Supplementary Figure 23. **Snapshot of the entire simulation cell of the J5 junction**. The HJ is shown with a colored surface surrounded by the truncated octahedron water box shown in grey (left). During the MD simulations which utilize the periodic boundary condition, the primary simulation cell is surrounded on all sides by its identical copies, forming an infinite reciprocal space. The periodic copies of the HJ and of the water are shown as blue surfaces and grey points, respectively. The position of the primary simulation cell within the periodic space is indicated with black lines.

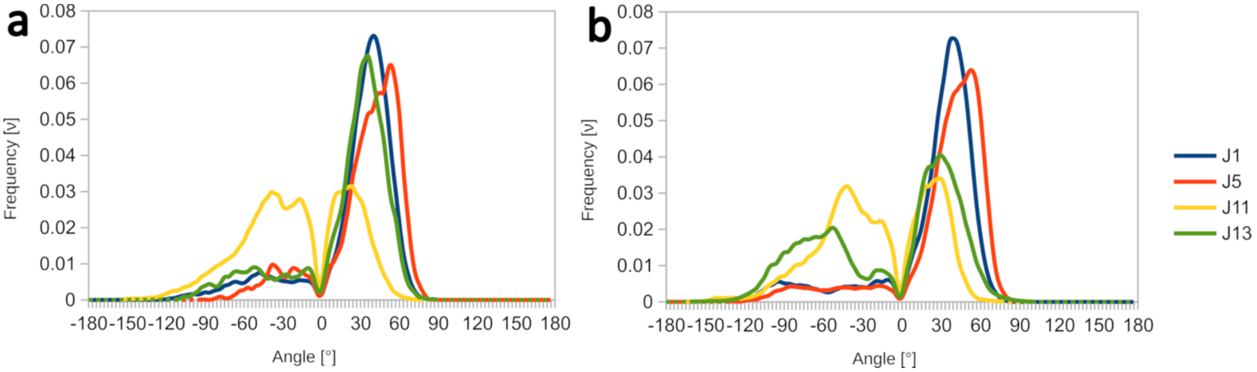

Supplementary Figure 24. **Comparison between standard- and extended-length MD simulations.** (a) Histograms of J_{twist} interhelical angle populations in MD simulations of J1, J5, J11, and J13 junctions which were run for one-microsecond (left); or (b) for twenty-microseconds (right). The populations of interhelical angles were very similar on both timescales, suggesting a relatively sufficient convergence of this structural parameter was achieved on the standard one-microsecond timescale.

Supplementary Table 20. PDB accession codes for each deposited structure.

	4x5		4x6 ($P3_2$)		4x6 ($R3$)		4x6 Scramble	
	Duplex	Junction	Duplex	Junction	Duplex	Junction	Duplex	Junction
1	5KEK	6X8C	5VY6	6XNA	—	—	7JKD	7JK0
2	—	—	7JPB	7JFT	—	—	7JKE	7JJZ
3	6WQG	6XDV	—	—	—	—	7JKG	7JJY
4	—	—	—	—	7JRY	7JHR	—	—
5	6WRB	6XDW	7JPA	7JFU	7JRZ	7JHS	7JKH	7JJX
6	6X8B	6XDX	—	—	—	—	—	—
7	6WSN	6XDY	7JPC	7JFV	—	—	7JKI	7JJW
8	6WSO	6XDZ	7JP9	6XO5	—	—	7JKJ	7JJ6
9	6WSP	6XEI	—	—	—	—	—	—
10	6WSQ	6XEJ	7JP8	7JFW	—	—	7JKK	7JJ5
11	—	—	—	—	—	—	—	—
12	—	—	—	—	—	—	—	—
13	—	—	—	—	—	—	—	—
14	6WSR	6XEK	—	—	—	—	7JL9	7JJ4
15	6WSS	6XEL	—	—	—	—	—	—
16	6WST	6XEM	7JP7	7JFX	—	—	7JLA	7JJ3
17	—	—	—	—	—	—	—	—
18	—	—	—	—	—	—	—	—
19	6WSU	6XFC	—	—	—	—	7JLB	7JJ2
20	6WSV	6XFD	7JP6	7JH8	—	—	—	—
21	6WSW	6XFE	—	—	—	—	7JLC	7JIQ
22	6WSX	6XFF	7JP5	7JH9	—	—	7JLD	7JIP
23	6WSY	6XFG	7JON	7JHA	—	—	7JLE	7JIO
24	6WSZ	6XFW	7JOL	7JHB	—	—	7JLF	7JIN
25	6WT0	6XGM	—	—	—	—	—	—
26	6WRJ	6XFX	7JOK	7JHC	—	—	7JNJ	7JIM
27	—	—	—	—	—	—	—	—
28	6WRI	6XFY	7JOJ	6XO6	—	—	—	—
29	6WT1	6XFZ	—	—	—	—	—	—
30	—	—	7JOI	6XO7	—	—	7JSB	7JI9
31	6WRC	6XG0	7JOH	6XO8	7JS0	7JHT	7JSC	7JI8
32	6WR9	6XGJ	—	—	—	—	—	—
33	6WR7	6XGN	7JOG	6XO9	7JS1	7JHU	7JNK	7JI7
34	6WRA	6XGO	—	—	—	—	7JNL	7JI6
35	6WR5	6XGK	—	—	—	—	—	—
36	6WR3	6XGL	—	—	7JS2	7JHV	7JNM	7JI5

Supplementary References:

1 Gelpí, J. L. *et al.* Classical molecular interaction potentials: Improved setup procedure in molecular dynamics simulations of proteins. *Proteins, structure, function, and bioinformatics* **45**, 428-437, doi:10.1002/prot.1159 (2001).

2 Liebl, K. & Zacharias, M. Tumuc1: A New Accurate DNA Force Field Consistent with High-Level Quantum Chemistry. *Journal of chemical theory and computation* **17**, 7096-7105, doi:10.1021/acs.jctc.1c00682 (2021).
