# Supplementary material for: The influence of Holliday junction sequence and dynamics on DNA crystal self-assembly
Source: Nat Commun. 2022 Jun 3;13:3112. doi: 10.1038/s41467-022-30779-6 (PMC9166708; doi:10.1038/s41467-022-30779-6)
Supplement: Supplementary file 2 — Description of Additional Supplementary Files [file 41467_2022_30779_MOESM2_ESM.pdf]

Description of Additional Supplementary Files

Supplementary Data 1: DNA sequences used for each constituent oligonucleotide combination for all 36 immobile junctions in each of the three systems used in this work.
