# Supplementary material for: The influence of Holliday junction sequence and dynamics on DNA crystal self-assembly
Source: Nat Commun. 2022 Jun 3;13:3112. doi: 10.1038/s41467-022-30779-6 (PMC9166708; doi:10.1038/s41467-022-30779-6)
Supplement: Supplementary file 4 — Reporting summary [file 41467_2022_30779_MOESM4_ESM.pdf]

nature research

Corresponding author(s): Hao Yan, Nicholas Stephanopoulos

Last updated by author(s): February 18, 2022

# Reporting Summary

Nature Research wishes to improve the reproducibility of the work that we publish. This form provides structure for consistency and transparency in reporting. For further information on Nature Research policies, see our Editorial Policies and the Editorial Policy Checklist.

## Statistics

For all statistical analyses, confirm that the following items are present in the figure legend, table legend, main text, or Methods section.

| n/a | Confirmed | |
|---|---|---|
| ☐ | ☒ | The exact sample size (*n*) for each experimental group/condition, given as a discrete number and unit of measurement |
| ☐ | ☒ | A statement on whether measurements were taken from distinct samples or whether the same sample was measured repeatedly |
| ☒ | ☐ | The statistical test(s) used AND whether they are one- or two-sided<br>*Only common tests should be described solely by name; describe more complex techniques in the Methods section.* |
| ☒ | ☐ | A description of all covariates tested |
| ☒ | ☐ | A description of any assumptions or corrections, such as tests of normality and adjustment for multiple comparisons |
| ☐ | ☒ | A full description of the statistical parameters including central tendency (e.g. means) or other basic estimates (e.g. regression coefficient) AND variation (e.g. standard deviation) or associated estimates of uncertainty (e.g. confidence intervals) |
| ☒ | ☐ | For null hypothesis testing, the test statistic (e.g. $F$, $t$, $r$) with confidence intervals, effect sizes, degrees of freedom and $P$ value noted<br>*Give P values as exact values whenever suitable.* |
| ☒ | ☐ | For Bayesian analysis, information on the choice of priors and Markov chain Monte Carlo settings |
| ☒ | ☐ | For hierarchical and complex designs, identification of the appropriate level for tests and full reporting of outcomes |
| ☒ | ☐ | Estimates of effect sizes (e.g. Cohen's *d*, Pearson's *r*), indicating how they were calculated |

*Our web collection on statistics for biologists contains articles on many of the points above.*

## Software and code

Policy information about availability of computer code

| | |
|---|---|
| Data collection | Standard synchrotron data collection methods were used at the Advanced Light Source (ALS), Argonne Photon Source (APS), and the National Synchrotron Light Source (NSLS-II) |
| Data analysis | HKL 2 000(v. 716.1) was used to process all crystallographic data, the PHENIX (v. 1.11.1-2575) and CCP4 (v. 6.5.016) packages were used for structure solution and refinement,along with model building in Coot (0.8.1 EL),Figures were prepared in PyMOL (v. 2.2.3) and all structures have been deposited to the PDB and have been released to the public. 3DNA-DSSR was used for analysis and calculation of the junction angles. AMBER18 (update.14) and VMD (v. 1.9.2) were used for MD simulations. |

For manuscripts utilizing custom algorithms or software that are central to the research but not yet described in published literature, software must be made available to editors and reviewers. We strongly encourage code deposition in a community repository (e.g. GitHub). See the Nature Research guidelines for submitting code & software for further information.

## Data

Policy information about availability of data

All manuscripts must include a data availability statement. This statement should provide the following information, where applicable:

- Accession codes, unique identifiers, or web links for publicly available datasets
- A list of figures that have associated raw data
- A description of any restrictions on data availability

All raw and processed data are available as indicated in the text. All coordinates and structure factors have been deposited and released to the public by the PDB. All corresponding accession codes can be found in the table in the Supplementary Information and are linked in the main text. All MD information is also publicly available as indicated.

# Field-specific reporting

Please select the one below that is the best fit for your research. If you are not sure, read the appropriate sections before making your selection.

☒ Life sciences ☐ Behavioural & social sciences ☐ Ecological, evolutionary & environmental sciences

For a reference copy of the document with all sections, see nature.com/documents/nr-reporting-summary-flat.pdf

# Life sciences study design

All studies must disclose on these points even when the disclosure is negative.

**Sample size** All statistical analyses and standard deviation calculations were based upon the raw values given in the tables in the SI.

**Data exclusions** No data were excluded.

**Replication** Calculations were based upon the final structure solution and refinement of the deposited structures.

**Randomization** This is not applicable to a structure study.

**Blinding** Blinding is not appropriate for this crystallographic and molecular simulation study. The statistics in the work were simply derived from the angles between junction arms of the crystal structures to probe variability between them. No other statistical analyses were performed to derive overall conclusions in the work.

# Reporting for specific materials, systems and methods

We require information from authors about some types of materials, experimental systems and methods used in many studies. Here, indicate whether each material, system or method listed is relevant to your study. If you are not sure if a list item applies to your research, read the appropriate section before selecting a response.

## Materials & experimental systems

| n/a | Involved in the study |
|---|---|
| ☒ | ☐ Antibodies |
| ☒ | ☐ Eukaryotic cell lines |
| ☒ | ☐ Palaeontology and archaeology |
| ☒ | ☐ Animals and other organisms |
| ☒ | ☐ Human research participants |
| ☒ | ☐ Clinical data |
| ☒ | ☐ Dual use research of concern |

## Methods

| n/a | Involved in the study |
|---|---|
| ☒ | ☐ ChIP-seq |
| ☒ | ☐ Flow cytometry |
| ☒ | ☐ MRI-based neuroimaging |
